# Electrochemically Deposited Ag/PANI on ITO: Non-Monotonic Disorder–Dispersion Coupling and Enhanced Third-Order Optical Nonlinearity

**DOI:** 10.3390/polym18070864

**Published:** 2026-03-31

**Authors:** Mahmoud AlGharram, Tariq AlZoubi, Yahia Makableh, Omar Mouhtady

**Affiliations:** 1 Department of Physics, School of Computing (SC), German Jordanian University, Amman 11180, Jordan; 2 College of Engineering and Technology, American University of the Middle East, Egaila 54200, Kuwait; yahia.makableh@aum.edu.kw (Y.M.); omar.mouhtady@aum.edu.kw (O.M.)

**Keywords:** Ag nanoparticles, indirect optical bandgap tuning, PANI, third-order optical nonlinearity, dielectric dispersion and free-carrier response

## Abstract

Conducting polymer–metal nanocomposites are widely investigated as tunable photonic and optoelectronic media; however, reported property trends often remain empirical because electronic disorder at the absorption edge, refractive-index dispersion, free carrier dielectric response, and third-order nonlinearity are rarely quantified within a single, composition-controlled film series. This limitation is particularly relevant for electrochemically grown PANI coatings on transparent conductive substrates, where nanoparticle incorporation can simultaneously enhance polarization while introducing aggregation-driven heterogeneity. Here, Ag/PANI nanocomposite thin films were fabricated directly on indium tin oxide (ITO) by potentiostatic electrodeposition from an aniline/camphorsulfonic acid electrolyte containing controlled Ag nanoparticle loadings (5–15 wt.%). This study addresses the research gap by integrating complementary optical-disorder and dispersion formalisms with dielectric and nonlinear analyses to establish a composition structure optics map for device-relevant films. Ag incorporation narrows the indirect optical gap from 1.98 eV (PANI) to 1.81 eV (5 wt.%), 1.38 eV (10 wt.%), and 1.19 eV (15 wt.%), while markedly broadening the Urbach tail (0.377 eV → 1.28–1.64 eV at 5–10 wt.%). Wemple–DiDomenico modeling and Drude-type dielectric dispersion reveal strongly non-monotonic evolution of oscillator energetics and the carrier response, culminating in large bound-electron dielectric constants (*ε_∞_* up to 469.8) and plasma frequencies (*ω_p_* up to 248 × 10^12^ Hz) at 15 wt.% Ag. Third-order nonlinearity is substantially enhanced but composition-sensitive: *χ*^3^ increases from 6.73 × 10^−9^ esu (PANI) to ~7.6 × 10^−8^ esu at 5 and 15 wt.%, whereas the Kerr coefficient peaks at 25.91 × 10^−7^ esu for 5 wt.% and is suppressed at intermediate/high loading. These results demonstrate that the optimal nonlinear performance is governed by a disorder–dispersion balance and microstructure-dependent local-field effects rather than the Ag fraction alone.

## 1. Introduction

Conducting polymer–metal nanocomposites have emerged as adaptable thin-film platforms in which electronic transport, dielectric polarization, and light–matter interaction can be jointly engineered through interfacial design [1]. By embedding metallic nanoparticles within a π-conjugated host, these hybrids combine the chemical tunability and low-temperature processability of polymers with the strong electronic polarizability and free-carrier response of metals. Such coupling is particularly attractive for optoelectronic components that demand simultaneous control over optical attenuation, refractive-index dispersion, and nonlinear susceptibility, including photodetectors, electro-optic modulators, optical limiters, and dielectric/optical sensors [2,3].

Among intrinsically conducting polymers, polyaniline (PANI) remains a canonical candidate because its electronic structure can be modulated by protonation and the redox state, enabling large changes in conductivity and optical response without altering the polymer backbone. Nevertheless, pristine PANI films typically exhibit limited optical density and a modest nonlinear performance in the near-infrared, while their dielectric response is strongly influenced by the microstructure and disorder. Incorporation of metallic nanophases offers a route to overcome these limitations. In particular, silver nanoparticles (Ag NPs) can introduce pronounced electronic polarization and broadband extinction through a combination of localized surface plasmon effects, interfacial charge transfer, and enhanced carrier density [4,5,6,7]. When appropriately dispersed, Ag NPs may also intensify local electromagnetic fields within the polymer matrix, thereby amplifying third-order nonlinearities relevant to Kerr-type refractive switching and optical limiting. At the same time, increasing metal loading can induce competing effects’ aggregation, percolation transitions, and defect-state formation that reshape the band-edge density of states and the dielectric loss landscape. Consequently, the functional outcome is not guaranteed to scale monotonically with filler fraction, and predictive design requires composition-resolved structure property correlations [8,9].

A substantial body of prior work has examined Ag-PANI composites for applications such as sensing, electromagnetic shielding, and conductivity enhancement. However, the optoelectronic literature is comparatively fragmented in two important respects. First, many studies report optical bandgap shifts or refractive-index changes without a unified analysis that connects band-edge tailing (disorder/defect states), dispersion energetics, and free-carrier dielectric behavior within a single materials series [10,11,12]. Second, third-order nonlinear optical parameters (e.g., *χ*^3^ and the nonlinear refractive index *n*_2_) are often reported either indirectly or in isolation, with limited discussion of how they coevolve with dispersion descriptors (such as Wemple–DiDomenico oscillator parameters) and dielectric loss mechanisms. This leaves a clear gap: a need for a systematic, composition-dependent investigation that links Ag incorporation to (***i***) bandgap narrowing and Urbach tail evolution, (***ii***) refractive-index dispersion and derived dielectric functions, (***iii***) free-carrier contributions in the long-wavelength regime, and (***iv***) the resulting third-order nonlinear optical response ideally for device-relevant thin films grown directly on transparent conductive substrates [13,14,15,16,17,18,19,20,21].

Previous nonlinear-optical studies on Ag/PANI systems have generally shown that the incorporation of Ag nanostructures enhances the third-order response of PANI by increasing local electromagnetic fields, interfacial polarization, and charge-transfer-assisted delocalization. However, most of these reports were limited to solution-processed composites or cast films and mainly emphasized isolated parameters such as optical limiting, nonlinear absorption, or *χ*^3^, without systematically relating them to band-edge disorder, refractive-index dispersion, and dielectric behavior. A similar trend is evident in broader PANI–metal nanocomposite benchmarks, where fillers such as Au, Ag, Cu, and other metallic nanophases improve polarizability and optical nonlinearity, but the magnitude of enhancement depends strongly on the nanoparticle loading, dispersion quality, interfacial coupling, and aggregation state. These benchmark studies collectively indicate that nonlinear enhancement in PANI–metal systems is not governed by the metal content alone; rather, it emerges from a balance between plasmonic/local-field amplification and microstructure-induced disorder. This underscores the need for composition-resolved studies that compare the nonlinear performance with dispersion and disorder descriptors within the same Ag/PANI thin-film series.

Electrochemical deposition provides a compelling pathway to address this gap. Compared with solution casting or ex situ blending, potentiostatic electropolymerization can yield conformal PANI coatings with strong adhesion and controllable thickness on conducting substrates, while simultaneously enabling in situ incorporation of nanoparticles from the electrolyte. Direct growth on indium tin oxide (ITO) is particularly advantageous for optoelectronic integration because it couples optical transparency with electrical addressability. Yet, despite these advantages, electrochemically deposited Ag/PANI thin films have not been comprehensively mapped in terms of composition–dispersion–nonlinearity relationships across a controlled Ag loading window. Establishing such relationships is essential for determining whether a given Ag fraction optimizes Kerr nonlinearity, whether band-edge disorder dominates absorption tailing, and how free-carrier polarization reshapes the dielectric spectrum. More broadly, nonlinear-optical enhancement is not limited to compositionally tuned nanocomposites; recent studies have shown that the metasurface and nonlocal meta-optical platforms can greatly amplify nonlinear responses through resonant field confinement, high-Q mode engineering, and tailored wavefront control. In this context, the present Ag/PANI films represent a materials-based route to nonlinear enhancement, where the governing factors are interfacial polarization, charge-transfer-assisted polarizability, and morphology-dependent local-field effects rather than lithographically defined metasurface resonances [22,23].

In this work, Ag/PANI nanocomposite thin films are fabricated by potentiostatic electrodeposition from an aniline/camphorsulfonic acid electrolyte containing controlled Ag NP loadings (5–15 wt.% relative to monomer), yielding uniform films deposited directly on ITO. The central novelty of this study is not merely the preparation of Ag/PANI films, but the integrated, composition-resolved evaluation of their electronic structure and optical response using complementary dispersion and disorder frameworks. Specifically, bandgap evolution is quantified through Tauc analysis alongside Urbach-tail extraction to capture how Ag incorporation modifies the band-edge density of states and electron–phonon coupling signatures. Refractive-index dispersion is interpreted via the Wemple–DiDomenico single-oscillator formalism to extract oscillator energetics and dispersion strength, enabling the comparison of linear polarizability trends across compositions. The dielectric function is then derived from optical constants to assess how polarization storage and loss channels evolve with the wavelength, while the infrared dielectric dispersion is further interpreted using a Drude-type (Spitzer-Fan) approach to elucidate free-carrier contributions. Finally, third-order nonlinear susceptibility and Kerr coefficients are evaluated within a consistent analytical scheme, enabling direct correlation between dispersion parameters, dielectric behavior, and nonlinear optical performance. This combined methodology exposes non-monotonic composition dependencies as an important outcome for rational design by showing that increasing the Ag fraction does not necessarily yield proportional increases in dispersion strength or nonlinear refraction [22,23,24,25,26,27,28].

The objectives of this study are therefore to (1) synthesize electrochemically deposited Ag/PANI nanocomposite thin films on ITO with controlled Ag NP loading; (2) establish composition-dependent trends in optical absorption, bandgap narrowing, and Urbach tailing as signatures of defect/disorder evolution; (3) quantify refractive-index dispersion and extract physically meaningful dispersion parameters to describe linear polarizability changes; (4) determine wavelength-dependent dielectric functions and identify the role of free carriers in long-wavelength dielectric dispersion; and (5) evaluate third-order nonlinear optical parameters and relate their enhancement (or suppression) to the underlying dispersion and microstructural effects. Collectively, these aims provide a structure–optics–nonlinearity map for electrochemically grown Ag/PANI films, supporting their targeted deployment in tunable photonic and optoelectronic devices.

## 2. Experimental Methods

### 2.1. Chemicals

Aniline (C_6_H_5_NH_2_, Sigma-Aldrich Co. Ltd., St. Louis, MO, USA) was used as received without additional purification. Camphorsulfonic acid (CSA, analytical grade) served as the protonic dopant for the polymerization process. Indium tin oxide (ITO)-coated glass substrates (2.5 cm × 5.5 cm) were supplied by Ossila Ltd. (Sheffield, UK), Silver NPs (Ag NPs) with an average particle size of approximately 20 nm were obtained from US Research Nanomaterials Inc. (Houston, TX, USA). Prior to film growth, the ITO substrates were cleaned by sequential ultrasonication in deionized water, absolute ethanol, and acetone for 5 min in each solvent to remove residual contaminants, improve surface wettability, and promote uniform adhesion of the deposited thin films.

### 2.2. Thin-Film Depositions

PANI and Ag/PANI nanocomposite thin films were fabricated by potentiostatic electrochemical deposition from an aniline/(CSA) electrolyte. The precursor solution contained 0.25 M aniline and 0.40 M CSA, corresponding to a CSA:aniline molar ratio of 1.6:1. This composition was selected to promote efficient protonation of the growing PANI chains, with CSA functioning both as the acidifying agent and as the dopant counter-ion. Throughout deposition, the electrolyte acidity was maintained at pH ≈ 1.2 to support electrochemically driven oxidative polymerization. After preparation, the aniline/CSA solution was portioned into equal aliquots, and each aliquot was supplemented with a predetermined mass of Ag nanoparticles (Ag NPs). The dispersions were homogenized by vortex mixing for 10 min to minimize particle agglomeration and improve uniformity. Three Ag loadings of 5, 10, and 15 wt.% relative to the aniline monomer were investigated. Electropolymerization was performed in a single-compartment cell at ~25 °C. A stainless-steel counter electrode and an ITO-coated glass working electrode were aligned parallel to each other and separated by a Teflon spacer, defining a 1 cm interelectrode gap. Film growth was initiated by applying a constant potential of 1.3 V for 3 min, yielding PANI and Ag/PANI coatings directly on the ITO substrates. While CSA served as the dopant during deposition, the resulting doping level in the films was not quantified. Film thickness, determined by optical profilometry, was approximately 1500 nm on average. For each composition, optical and electrical measurements were performed on three independently prepared samples, and the reported values represent the mean of these replicates.

### 2.3. Evaluation of Optical and Structural Properties

The optical response of the deposited films was characterized using a Shimadzu UV–Vis spectrophotometer (UV-100). Normal-incidence transmittance, *T*(*λ*), was collected over 200–3300 nm and converted to the absorption coefficient (*α*) using

(1) $$\alpha =-\ln\left(T\right)/t$$

where *t* is the film thickness (nm). The extinction coefficient (*k*) was subsequently calculated from *α* according to

(2) $$k=\frac{\alpha \lambda }{4\pi }\;$$

with *λ* denoting the incident wavelength (nm). The real refractive index (*n*), which governs the phase velocity of light within the absorbing film, was determined from the measured reflectance (*R*) together with *k* via

(3) $$n=\left(\frac{1+R}{1-R}\right)-\sqrt{\frac{4R\;}{{\left(1-R\right)}^{2}}-k^{2}}$$

Equation (3) corresponds to the physically admissible branch for absorbing thin films. The crystalline structure was examined by X-ray diffraction (XRD) using a Shimadzu 7000 diffractometer (Kyoto, Japan) with Cu *K_α_* radiation (*λ* = 1.5406 Å) operated at 40 kV and 40 mA. The average crystallite size and microstrain were estimated using the Williamson–Hall method, which deconvolutes peak broadening into size- and strain-related contributions to provide representative crystallite dimensions and internal lattice strain in the nanocomposite films. Fourier-transform infrared (FTIR) spectra were acquired on a PerkinElmer instrument in the 4000–400 cm^−1^ range to assign characteristic vibrational bands and to assess chemical interactions between the PANI matrix and the embedded nanoparticles. The refractive-index dispersion and nonlinear optical response were analyzed within the Wemple–DiDomenico single-effective-oscillator framework, which offers a compact description of *n*(*λ*) and enables the extraction of key dispersion parameters relevant to nonlinear-optical performance in optoelectronic applications. Surface morphology was evaluated by atomic force microscopy (AFM) in the tapping mode over a 20 µm × 20 µm scan area (512 × 512 pixels). Height maps were first-order plane-leveled and flattened line-by-line prior to quantitative processing. Areal roughness descriptors were including S_a_ (arithmetic mean height), S_q_ (root-mean-square height), and S_z_ (peak-to-valley height; R_z_ = z_max_ − z_min_). Values reported in Table A1 were extracted from the same scan regions displayed in the AFM images; S_a_, S_q_, and S_z_ are given in nm, with S_z_ additionally reported in µm.

## 3. Results and Discussion

### 3.1. Nanocomposites and Film Deposition

Prior to fabricating the nanocomposite coatings, the suitability of ITO substrates for PANI electropolymerization was first evaluated. Electrochemical experiments were performed in a conventional three-electrode cell, employing ITO-coated glass as the working electrode and an aniline/camphorsulfonic acid (CSA) solution as the precursor medium. The ITO electrode was placed in an acidic electrolyte containing the monomer–dopant mixture, enabling in situ oxidative polymerization of aniline directly onto the ITO surface. These preliminary measurements verified the electrochemical responsiveness of the aniline/CSA system on ITO and confirmed that the substrate provides an appropriate platform for subsequent growth of PANI-based nanocomposite thin films [26].

### 3.2. XRF Characterization

**Figure 1:**
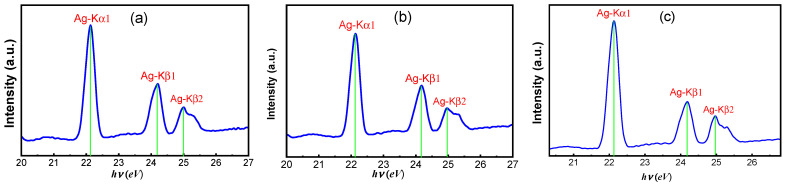
X-ray fluorescence (XRF) spectra of Ag/PANI nanocomposite thin films containing (**a**) 5 wt.%, (**b**) 10 wt.%, and (**c**) 15 wt.% Ag nanoparticles. Distinct Ag K lines are observed at ~22.16 keV (Kα1) and ~24.94 keV (Kβ1), with a Kβ2 feature near ~25.45 keV, confirming successful Ag NP incorporation.

X-ray fluorescence spectroscopy was employed to verify the elemental incorporation of Ag into the PANI matrix and to assess the compositional consistency of the Ag/PANI nanocomposite films. Figure 1 presents the XRF spectra in the photon-energy window of 20–27 keV for films prepared with 5, 10, and 15 wt.% Ag. All spectra are dominated by the characteristic Ag K-series emissions, demonstrating that Ag is present in each nanocomposite composition. Three Ag-related features are clearly resolved. The strongest peak occurs at *hν* ≈ 22.16 keV, assigned to the Ag-K_α1_ line. Two higher-energy peaks follow at *hν* ≈ 24.94 keV (Ag-K_β1_) and *hν* ≈ 25.45 keV (Ag-K_β2_). The separation between K_α1_ and K_β1_ is ≈ 2.78 keV, which matches the expected K-shell transition spacing for Ag and supports the correctness of the peak assignments. The relative line intensities increase with Ag loading, consistent with a higher Ag areal density in the film. Quantitatively, the maximum K_α1_ peak intensity rises from ≈1.10 a.u. for the 5 wt.% film (Figure 1a) to ≈1.35 a.u. for 10 wt.% (Figure 1b) and reaches ≈1.80 a.u. for 15 wt.% (Figure 1c). Using these maxima, the K_α1_ intensity increases by ≈0.25 a.u. from 5→10 wt.% and by ≈0.45 a.u. from 10→15 wt.%, giving an overall ΔI ≈ 0.70 a.u. across 5→15 wt.%. A similar, though weaker, loading dependence is observed for the Kβ_1_ line at ~24.94 keV. The Kβ_1_ peak increases from ≈0.55 a.u. (5 wt.%) to ≈0.70 a.u. (10 wt.%) and ≈0.70–0.75 a.u. (15 wt.%). Accordingly, the approximate intensity ratio I(K_β1_)/I(K_α1_) remains in the narrow range ≈0.39–0.50 (0.55/1.10 ≈ 0.50; 0.70/1.35 ≈ 0.52; 0.70/1.80 ≈ 0.39), indicating that the spectral shape is preserved while the overall signal scales with the Ag content [24,28]. The K_β2_ contribution near ~25.45 keV is weaker, reaching ≈0.35 a.u. (5–10 wt.%) and ≈0.45 a.u. (15 wt.%). Beyond the Ag K lines, no additional intense peaks are evident within 20–27 keV, suggesting that no high-Z contaminants contribute significantly in this energy interval. Although XRF in this window does not probe light elements (C, N, H) that form the PANI backbone, the clean Ag K-line signature and its systematic growth with loading provide strong evidence for successful incorporation of Ag nanoparticles and for a composition-dependent increase in Ag content in the deposited films. These XRF results support subsequent structure property correlations, particularly the monotonic enhancement observed in electrical conductivity with an increasing Ag fraction.

### 3.3. Atomic Force Microscopy (AFM) Characterization

**Figure 2:**
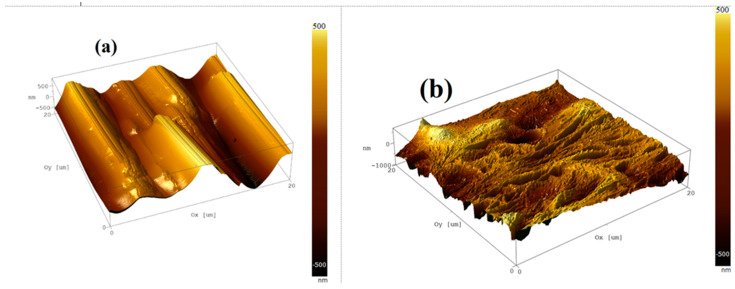
Three-dimensional AFM topography of the films acquired over a 20 × 20 μm^2^ area: (**a**) pristine PANI showing a smoother, wavy surface (R_a_ = 0.240088 μm, R_q_ = 0.303869 μm; surface area = 484.02 μm^2^ vs. projected area = 398.439 μm^2^), and (**b**) Ag/PANI at 15 wt.%, with the nanocomposite film showing a rougher morphology (R_a_ = 0.637 μm, R_q_ = 0.830 μm, R_z_ ≈ 4.74 μm; surface area = 517.3 μm^2^ vs. projected area = 398.4 μm^2^, ~30% increase).

**Figure A1:**
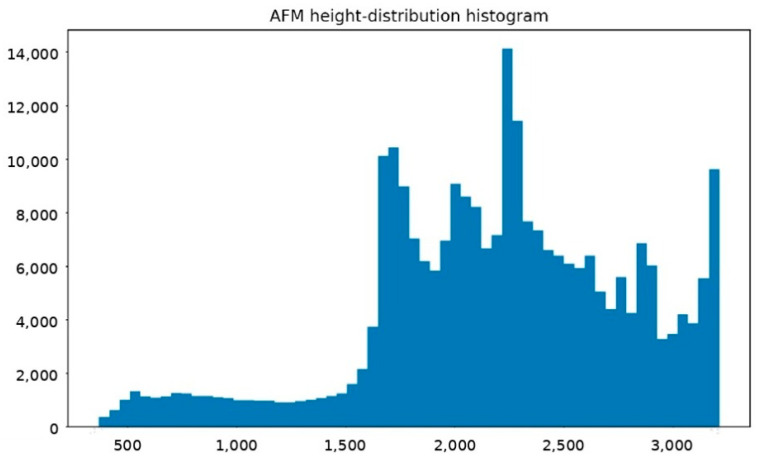
AFM height-distribution histogram of the pristine PANI film recorded over a 20 × 20 µm^2^ scan area. The histogram represents the statistical distribution of height values across the scanned surface and provides quantitative insight into the topographic uniformity and roughness of the polymer film.

**Figure A2:**
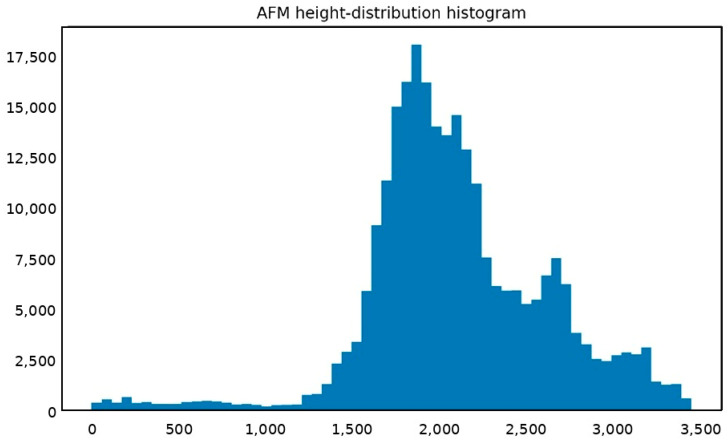
AFM height-distribution histogram of the Ag/PANI (15 wt.%) nanocomposite film recorded over a 20 × 20 µm^2^ scan area. The histogram shows the distribution of surface–height values extracted from the AFM topography image, reflecting the surface roughness and height heterogeneity of the film.

Figure 2 presents three-dimensional AFM topographies acquired over a 20 × 20 μm^2^ region for (a) the pristine PANI and (b) Ag/PANI at 15 wt.%, the nanocomposite film. The pristine PANI exhibits a comparatively smooth, wavy relief with limited sharp features, indicating a more uniform growth front and fewer abrupt height discontinuities. Quantitatively, the pristine surface yields Ra = 0.240088 μm and Rq = 0.303869 μm, with height extrema of +1.13055 μm (maximum) and −1.15284 μm (minimum). The near-zero skewness (−0.000482) suggests an almost symmetric distribution of peaks and valleys around the mean plane, while the slightly negative kurtosis (−0.0756521) implies a modestly flatter than Gaussian height distribution consistent with a surface dominated by broad undulations rather than isolated spikes or pits. In contrast, the nanocomposite film displays a markedly rougher and more heterogeneous landscape, characterized by pronounced ridges and deeper valleys across the same lateral scale. This evolution is reflected in the substantial increase in roughness parameters to Ra = 0.637 μm and Rq = 0.830 μm, together with a large peak-to-valley measure of Rz ≈ 4.74 μm (range 2.389–7.130 μm). Such increases indicate that nanoparticle incorporation (and the associated changes in nucleation density, local conductivity, and mass transport during growth) intensifies vertical texture development, producing a more corrugated interface. Beyond roughness alone, AFM enables the estimation of the true surface area relative to the projected (planar) area, which is critical when performance depends on the real contact area. For the pristine film, the true surface area is 484.02 μm^2^ versus a projected area of 398.439 μm^2^, corresponding to an area increase of ≈21.5%. The nanocomposite film shows an even larger enlargement: 517.3 μm^2^ true surface area compared with 398.4 μm^2^ projected area, i.e., ≈29.8% increase (∼30%). This measurable gain in effective area is expected to (i) increase the density of accessible adsorption/interaction sites, (ii) enhance electrode–electrolyte contact for electrochemical processes, and (iii) promote mechanical interlocking with overlayers or substrates. At the same time, the strong rise in Rq and Rz indicates more severe height excursions, which can introduce local field concentration and nonuniform current pathways; depending on the target application, this may be beneficial (more active sites, improved coupling) or detrimental (greater scattering, localized defects, and percolation inhomogeneity). Overall, the AFM results demonstrate a clear morphological transition from a relatively uniform, wave-like pristine surface to a significantly more textured nanocomposite surface with a higher surface-area enlargement factor. This provides a quantitative microstructural basis for interpreting any concurrent changes in optical response, charge transport, and interfacial sensitivity reported in subsequent sections. Beyond the morphological description, the AFM results provide a microstructural basis for interpreting the optical and nonlinear response of the films. The progressive increase in surface roughness and surface-area development with Ag incorporation is expected to enhance interfacial polarization, increase local electromagnetic field concentration, and introduce greater surface heterogeneity, all of which influence light–matter interaction within the nanocomposite layer. These morphology-driven changes can therefore contribute to the observed variations in refractive-index dispersion, dielectric behavior, and third-order nonlinear optical parameters. Accordingly, the AFM-derived roughness evolution is not merely descriptive, but is physically consistent with the composition-dependent enhancement in the optical and nonlinear performance of the Ag/PANI films. In addition to the three-dimensional AFM topographies, height-distribution histograms were included to provide a statistical representation of the surface–height variation across the scanned area (Figure A1 and Figure A2). These histograms complement the roughness parameters by illustrating the spread, symmetry, and dominant height ranges of the surface features. The broader and more irregular height distribution observed for the Ag-containing films indicates increased surface heterogeneity and more pronounced vertical relief compared with pristine PANI, in agreement with the roughness evolution derived from the AFM analysis. The observed morphological evolution is closely linked to the optical and nonlinear behavior of the Ag/PANI films. With increasing Ag incorporation, the AFM results reveal a progressive increase in surface roughness, granular development, and microstructural heterogeneity, indicating a more complex and highly textured nanocomposite surface. Such changes can enhance interfacial polarization and local field concentration at the Ag/PANI boundaries, while also modifying the effective optical path and light–matter interaction within the film. Consequently, the morphology development provides a structural basis for the observed variations in refractive-index dispersion, dielectric response, optical conductivity, absorption-edge broadening, and third-order nonlinear optical parameters. Therefore, the composition-dependent optical and nonlinear performance of the films can be understood not only in terms of Ag content but also through the accompanying evolution of the nanocomposite microstructure.

### 3.4. Optical Properties

**Figure 3:**
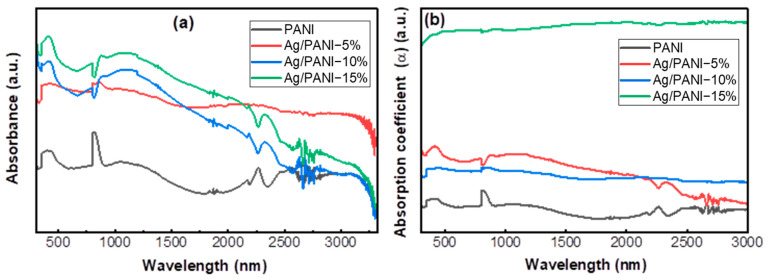
(**a**) Absorbance spectra and (**b**) absorption coefficients for the respective materials.

Figure 3a shows the UV-Vis-NIR absorbance spectra of pristine PANI and Ag/PANI nanocomposite films (5, 10, and 15%) measured over approximately 359–3354 nm. Pristine PANI exhibits comparatively weak absorption with a visible band centered at 450.091 nm (A = 0.293) and a stronger near-IR feature at 867.514 nm (A = 0.333). In the extended near-IR, PANI displays only modest shoulders/maxima at 2305.808 nm (A = 0.210), 2614.338 nm (A = 0.226), and 2777.677 nm (A = 0.221), consistent with a broad polaronic/free-carrier background rather than sharp electronic transitions. Representative absorbance values for PANI are A (500 nm) = 0.278, A (1000 nm) = 0.239, A (2000 nm) = 0.127, A (3000 nm) = 0.203, and A (3300 nm) = 0.096. Incorporation of Ag nanoparticles markedly amplifies the absorbance across the entire spectral window, confirming a substantial increase in optical extinction (absorption and additional loss mechanisms) relative to the pristine polymer. The enhancement is most pronounced in the visible region: at 450.091 nm, the absorbance increases from 0.293 (PANI) to 0.563 (Ag/PANI-5%), 0.650 (Ag/PANI-10%), and 0.748 (Ag/PANI-15%), corresponding to relative gains of +91.9%, +121.6%, and +155.1%, respectively. The Ag/PANI-15% film reaches the highest overall absorbance in the dataset at 450.091 nm (A = 0.748), while Ag/PANI-10% peaks at the same wavelength with A = 0.650, indicating that higher Ag loading intensifies visible-light attenuation. A pronounced loading-dependent evolution is also observed in the near-IR, where the broad carrier-related band strengthens and shifts toward longer wavelengths. Ag/PANI-5% shows its dominant maximum at 894.737 nm (A = 0.567), whereas Ag/PANI-10% and Ag/PANI-15% develop stronger bands at 1130.672 nm (A = 0.621) and 1117.060 nm (A = 0.687), respectively. At 1000 nm, absorbance increases from 0.239 (PANI) to 0.542 (5%), 0.600 (10%), and 0.674 (15%), i.e., enhancements of +127.2%, +151.6%, and +182.4%. This intensification and red-shift of the near-IR response are consistent with an increased density of delocalized charge carriers and stronger polaron/bipolaron contributions promoted by Ag-PANI interfacial interactions. In the long-wavelength region, the spectra reveal a non-monotonic tail behavior. At 2000 nm, the absorbance rises from 0.127 (PANI) to 0.476 (5%), 0.393 (10%), and 0.510 (15%), evidencing a significant growth of the low-energy absorption tail upon Ag incorporation. Beyond approximately 2400–2800 nm, Ag/PANI-10% and Ag/PANI-15% show more pronounced attenuation, reaching minima of 0.192 at 2723.230 nm (10%) and 0.268 at 2777.677 nm (15%), while Ag/PANI-5% remains comparatively elevated (0.438 at 2768.603 nm). Consequently, at 3300 nm, the absorbance follows the order 5% (0.389) > 15% (0.149) > 10% (0.111) > PANI (0.096), indicating that the 5% loading preserves the strongest far-NIR tail. Collectively, these trends demonstrate that Ag nanoparticles substantially enhance optical extinction in PANI, strengthen and red-shift the carrier-related near-IR band, and modify the sub-gap/low-energy absorption tail in a manner that depends on Ag loading, likely reflecting the combined roles of plasmon-related extinction, interfacial charge transfer, and microstructure/dispersion effects in the nanocomposite films [29].

Figure 3b compares the absorption coefficient spectra, *α* (a.u.), of pristine PANI and Ag/PANI nanocomposite films (5, 10, and 15%) across approximately 500–3000 nm. The absorption coefficient was obtained from the measured absorbance using the Beer–Lambert relation (Equation (4)).

*α* = 2.303*At*^−1^ (4)

where *A*(*λ*) is the absorbance at wavelength *λ*, and *t* is the film thickness. A clear Ag-loading dependence is evident: incorporation of Ag increases *α* over the full spectral window and progressively suppresses the wavelength selectivity of the polymer, yielding a more broadband optical response at a higher Ag content. For pristine PANI, α remains low throughout the spectrum and informs a weak near-IR tail. Quantitatively, *α* equals 0.126 at 500 nm and stays near 0.126 at 1000 nm, then decreases to 0.085 at 1500 nm and reaches 0.068 at 2000 nm. At longer wavelengths, the tail slightly recovers, giving 0.107 at 2500 nm and 0.103 at 3000 nm. The main local maximum occurs at around 834 nm, where *α* reaches 0.170, while the lowest region is centered near ~1794 nm with α = 0.061. Upon adding 5% Ag, *α* increases strongly in the visible-near-IR and then gradually declines toward longer wavelengths. At 500 nm, α rises to 0.341 (vs. 0.126 for PANI) and remains high at 1000 nm with *α* = 0.349. The spectrum then decreases to 0.304 at 1500 nm and 0.257 at 2000 nm, followed by a more pronounced drop in the far-NIR to 0.167 at 2500 nm and 0.130 at 3000 nm. A local maximum is observed near ~418 nm with *α* = 0.383, and the response exhibits a broad plateau in the ~700–1200 nm interval (average *α* = 0.339). For 10% Ag, the spectrum becomes more uniform and maintains a moderate α level across the full range. The absorption coefficient is 0.286 at 500 nm, 0.279 at 1000 nm, 0.246 at 1500 nm, 0.242 at 2000 nm, 0.229 at 2500 nm, and 0.225 at 3000 nm, indicating only a gentle decay with the wavelength compared with the 5% film. In the visible region, a local high region occurs near ~411 nm with α ≈ 0.290, while the near-IR maximum appears at around ~857 nm with *α* = 0.291. The small change between 2000 and 3000 nm (0.242 to 0.225) indicates a stable long-wavelength tail for the 10% sample. The non-monotonic behavior of the 10 wt.% film indicates that the measured response is not controlled solely by Ag loading, but also by microstructural factors such as Ag distribution, partial clustering, and interfacial coupling within the PANI matrix. At this intermediate composition, these competing effects can produce a transitional response that deviates from the trend observed for the 5 and 15 wt.% films. The most striking behavior is observed for 15% Ag, which yields an almost wavelength-independent, high absorption coefficient across the entire measured range. The values are α = 0.899 (500 nm), 0.907 (1000 nm), 0.928 (1500 nm), 0.940 (2000 nm), 0.935 (2500 nm), and 0.942 (3000 nm). The average α increases systematically from 0.126 (PANI) to 0.339 (5%), 0.279 (10%), and 0.906 (15%) within 700–1200 nm, and from 0.081 (PANI) to 0.296 (5%), 0.248 (10%), and 0.929 (15%) within 1200–2000 nm, confirming strong broadening and amplification of optical attenuation with an increased Ag fraction. At 2000 nm, the 15% film (*α* = 0.940) exceeds pristine PANI (*α* = 0.068) by a factor of approximately 13.8 X. Overall, the spectra demonstrate that Ag addition increases the magnitude of *α* and reshapes its spectral profile: 5% Ag produces high visible-near-IR absorption followed by a pronounced far-NIR decline, 10% Ag stabilizes the long-wavelength tail at a moderate level, and 15% Ag drives the system into a regime of nearly constant, high *α* across 500–3000 nm. This evolution is consistent with an increased carrier density and interfacial charge transfer in Ag/PANI, together with broadband optical losses introduced by the metallic phase (including plasmon-related extinction and microstructure-dependent scattering), which become increasingly dominant at higher Ag loading [30].

**Figure 4:**
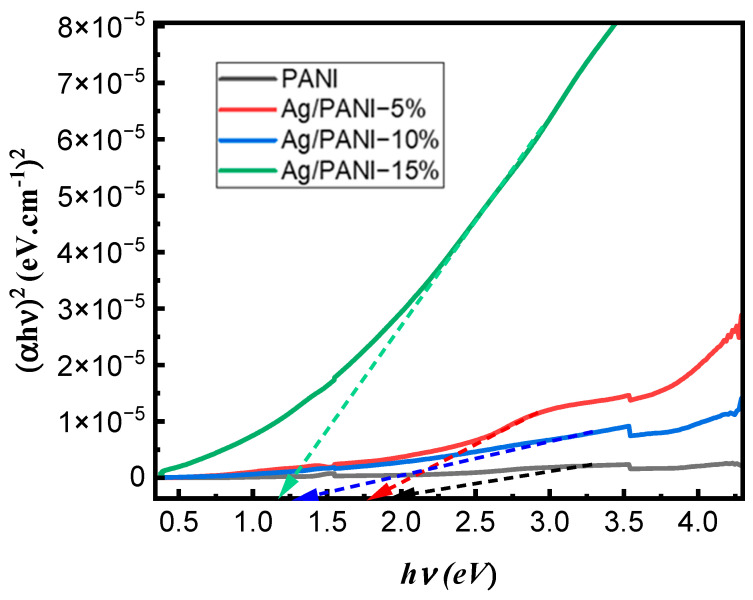
Analysis of (*αhv*)^2^ as a function of incident photon energy for PANI and nanocomposites.

Figure 4 presents the Tauc analysis of the optical transition for pristine PANI and Ag/PANI nanocomposite films (5, 10, and 15 wt.%) by plotting (*αhν*)^2^ as a function of photon energy (*hν*). The optical bandgap was extracted from the linear portion of each curve near the absorption edge by extrapolating the fitted straight line to the energy axis ((*αhν*)^2^ → 0), following the Tauc relation [30]:

(5) $${\left(\alpha h\nu \right)}^{1/n}=\;\beta (h\nu -E_{g})$$

where *α* is the absorption coefficient, *hν* is the photon energy, *β* is a proportionality constant, and *E_g_* is the optical energy gap. In the representation used in Figure 4, the ordinate is (*αhν*)^2^, corresponding to *n* = 1/2 in Equation (5). The Tauc plots exhibit a clear, Ag-loading-dependent displacement of the absorption edge toward lower photon energies, demonstrating progressive bandgap narrowing upon the incorporation of Ag nanoparticles. The extracted energy-gap values (Table 1) decrease systematically from *E_g_* = 1.98 eV for pristine PANI to 1.81 eV for Ag/PANI—5 wt.%, 1.38 eV for Ag/PANI—10 wt.%, and 1.19 eV for Ag/PANI—15 wt.%. Relative to pristine PANI, the absolute reductions are 0.17 eV (5 wt.%), 0.60 eV (10 wt.%), and 0.79 eV (15 wt.%), which correspond to decreases of 8.59%, 30.30%, and 39.90%, respectively. The largest incremental change occurs when increasing the Ag content from 5 to 10 wt.% (Δ*E_g_* = 0.43 eV, a 23.76% reduction relative to the 5 wt.% film), whereas the change from 10 to 15 wt.% is smaller (Δ*E_g_* = 0.19 eV, 13.77% relative to the 10 wt.% film). These quantitative trends are consistent with Ag-induced modification of the polymer matrix’s electronic structure. In particular, the observed bandgap narrowing, together with the broadening of the Urbach tail and the strengthening of low-energy optical absorption, suggests an increase in localized interfacial/disorder-related states near the band edge and a possible enhancement of carrier delocalization within the Ag/PANI nanocomposite. Therefore, this interpretation is inferred from the combined optical-disorder and conductivity trends rather than claimed as a direct electronic-structure measurement. In Ag/PANI, interfacial interactions between metallic nanoparticles and the π-conjugated backbone can promote charge transfer and increase the density of polaronic and bipolaronic states, effectively extending the absorption tail to lower energies and shifting the Tauc intercept to smaller *E_g_*. The monotonic narrowing from 1.98 to 1.19 eV therefore supports a transition from a wider gap polymer response to a more metallic/strongly doped electronic structure at higher Ag loading, consistent with the broadband optical strengthening observed in the corresponding absorbance and absorption-coefficient spectra [27,28,29,30,31,32,33].

**Figure 5:**
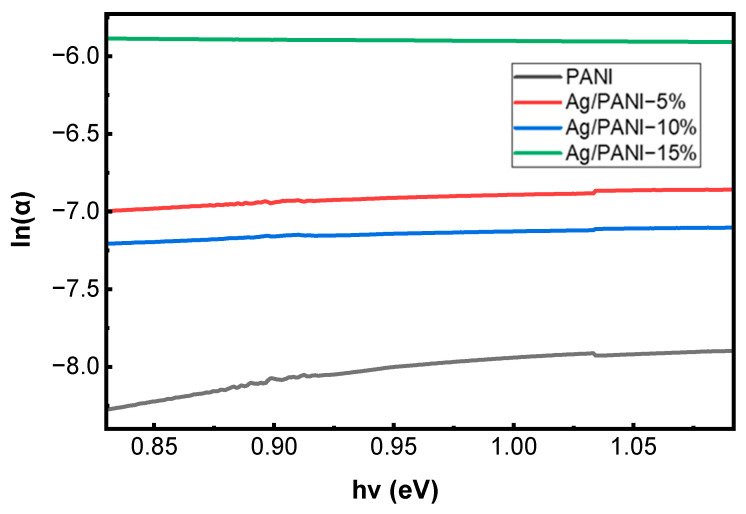
Urbach energy variation with incident photon energy in PANI and nanocomposites.

**Figure 6:**
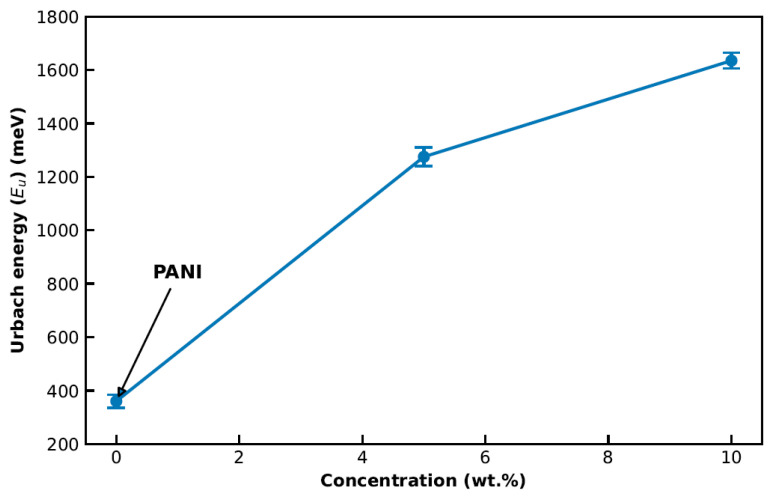
Variation in Urbach energy (*E_U_*) as a function of nanofiller concentration for PANI and its nanocomposites.

Figure 5 and Figure 6 summarize the Urbach-tail analysis for pristine PANI and Ag/PANI nanocomposite films (5, 10, and 15 wt.% Ag), and Table 1 lists the derived disorder- and coupling-related parameters. In the sub-bandgap (exponential) absorption region, the absorption coefficient follows the Urbach relation:

(6) $$\alpha \;=\;\alpha_{0}\;exp(hv/E_{U})$$

where *α*_0_ is a pre-exponential factor, *hν* is the photon energy, and *E_U_* is the Urbach energy. Taking the natural logarithm gives *ln*(*α*) = *ln*(*α*_0_) *+* (*hν/E_U_*), so *E_U_* is obtained from the inverse slope of the linear part of the *ln*(*α*) versus *hν* plot (Figure 5).

Figure 5 shows that pristine PANI exhibits the steepest *ln*(*α*) versus *hν* dependence (largest slope), consistent with the smallest Urbach energy and a relatively sharper absorption edge. Quantitatively, Table 1 yields *E_U_* = 377 meV (0.377 eV) for PANI. The corresponding Urbach slope is 1/*E_U_* = 2.652 eV^−1^, indicating a comparatively narrow distribution of localized tail states. Upon incorporating Ag, the *ln*(*α*) versus *hν* curves shift upward and become less steep, evidencing a broadening of the exponential tail. Specifically, *E_U_* increases to 1280 meV (1.280 eV) for Ag/PANI—5 wt.% and to 1640 meV (1.640 eV) for Ag/PANI—10 wt.% (Table 1), which reduces the Urbach slopes to 0.781 and 0.610 eV^−1^, respectively. Relative to pristine PANI, *E_U_* rises by +903 meV at 5 wt.% (an increase of 239.5%) and by +1263 meV at 10 wt.% (an increase of 335.0%). The incremental change from 5 to 10 wt.% is +360 meV (28.1%), confirming that the dominant broadening of the sub-gap tail occurs already at moderate Ag loading. Figure 6 visualizes the same trend by plotting *E_U_* versus nanofiller concentration: *E_U_* increases from 377 meV at 0 wt.% (PANI) to 1280 meV at 5 wt.% and 1640 meV at 10 wt.%, corresponding to ≈3.40× and ≈4.35× amplification relative to the pristine polymer. These increases indicate a substantial rise in structural/electronic disorder and a higher density of localized states in the gap region, which is consistent with the formation of additional tail states at Ag/PANI interfaces and with enhanced carrier localization arising from nanofiller-induced microstructural heterogeneity. The steepness parameter *γ* (*γ* = *k_B_ T/E_U_*) (eV^−1^) [31] (Table 1) decreases monotonically with Ag content, from 0.06 eV^−1^ for PANI to 0.02 eV^−1^ for 5 wt.% and 0.01 eV^−1^ for 10 wt.%, i.e., reductions by factors of 3 and 6 relative to pristine PANI. In parallel, the strength of electron–phonon interaction *E_e_p_* (*E_e_p_* = 2/3 *γ* [31]) increases from 10.7 eV (PANI) to 36.2 eV (5 wt.%) and 46.4 eV (10 wt.%), representing increases of 3.38× and 4.34×, respectively. The simultaneous increase in *E_U_* and *E_e_p_*, together with the decrease in *γ*, supports stronger coupling between electronic states and lattice/segmental vibrations and a broader energetic spread of localized states as the Ag concentration rises. For Ag/PANI—15 wt.%, Table 1 reports the Urbach parameters as “not reliable” (*E_U_*), with an undefined *γ* and *E_e_p_* (***). This limitation is consistent with the deviation from a well-defined linear Urbach regime in *ln*(*α*) versus *hν* at high loading, where strong metallic extinction, aggregation-related scattering, and/or saturation of sub-gap absorption can obscure the exponential tail needed for robust fitting. The lack of a reliable Urbach–energy fit for the 15 wt.% Ag/PANI film does not negate the overall disorder-related trend observed at lower Ag contents. Rather, it indicates that at the highest loading, the absorption-edge region becomes too complex to be adequately described by a single-exponential Urbach model. The progressive increase in *E*_*U*_ from pristine PANI to 5 wt.% and 10 wt.% already confirms substantial broadening of the absorption edge with Ag incorporation. For the 15 wt.% film, the fitting limitation is itself physically meaningful, as it suggests the coexistence of multiple loss contributions, including strong metallic extinction, interfacial absorption, aggregation-related scattering, and increased edge-state complexity. Therefore, this sample is better interpreted as marking the onset of a more optically complex regime, rather than providing a quantitatively reliable *E*_*U*_ value within the simple Urbach framework. Overall, the combined results in Figure 5 and Figure 6 and Table 1 demonstrate that Ag incorporation substantially broadens the Urbach tail (*E_U_*: 0.377 → 1.640 eV), reduces the absorption-edge steepness (*γ*: 0.06 → 0.01 eV^−1^), and strengthens electron–phonon interaction (*E_e_p_*: 10.7 → 46.4 eV), confirming a pronounced increase in disorder-driven tail states and interfacial coupling in the Ag/PANI nanocomposite system.

- **Optical constants: Extinction coefficient (*K*) and refractive index (*n*)**

**Figure 7:**
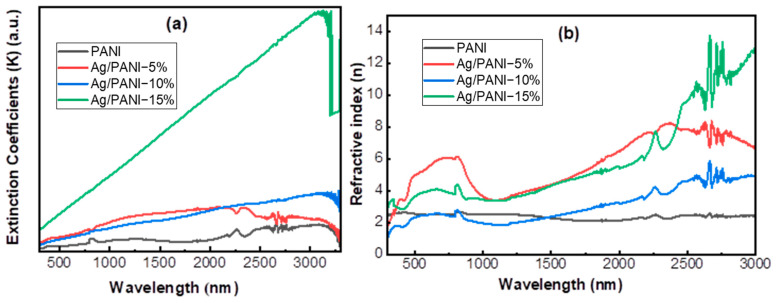
Optical constants of PANI and Ag/PANI (5–15 wt.%): (**a**) extinction coefficient (*K*) and (**b**) refractive index (*n*) as functions of wavelength, highlighting enhanced optical loss and stronger dispersion with higher Ag content.

Figure 7 presents the optical constants of pristine PANI and Ag/PANI nanocomposite films (5–15 wt.% Ag) as functions of wavelength, including (a) the extinction coefficient (*K*) and (b) the refractive index (*n*) over the visible-near-IR window (≈500–3000 nm). In both panels, Ag incorporation produces a marked strengthening of optical loss and dispersion, with the magnitude and wavelength dependence controlled by the Ag loading. Pristine PANI exhibits low *K* values throughout the spectrum, increasing from *K* (500 nm) = 0.010 to *K* (3000 nm) = 0.070, with a gradual rise in the long-wavelength region. Introducing Ag elevates *K* across the full window. At 500 nm, *K* increases to 0.036 (5%), 0.029 (10%), and 0.102 (15%), i.e., enhancements of 3.45×, 2.83×, and 9.86× relative to PANI. The Ag/PANI—5% film increases up to *K* = 0.120 at 2000 nm and then decreases to 0.089 at 3000 nm, indicating that optical loss peaks in the mid-IR/near-IR range for this loading [34,35].

In contrast, Ag/PANI—10% increases more monotonically from 0.029 (500 nm) to 0.159 (3000 nm), reaching a maximum *K* ≈ 0.164 near 3100 nm. The Ag/PANI—15% film shows the strongest and most rapidly rising loss, increasing from 0.102 at 500 nm to 0.674 at 3000 nm, with a maximum *K* ≈ 0.686 near 3078 nm; at 3000 nm, this corresponds to ≈9.65× the PANI value, evidencing a transition to a highly lossy optical response at high Ag content. Pristine PANI shows weak dispersion with *n* ≈ 2.52 at 500 nm, a shallow minimum *n* ≈ 2.07 near 1712 nm, and *n* ≈ 2.38 at 3000 nm, consistent with a relatively low-loss polymer matrix. Ag NP addition substantially raises *n* and amplifies its wavelength dependence. For Ag/PANI—5%, *n* increases from 4.912 at 500 nm to 6.014 at 800 nm, then drops to 3.603 at 1000 nm before rising to a maximum *n* ≈ 8.194 near 2354 nm and ending at *n* (3000 nm) = 6.706. The Ag/PANI—10% film exhibits a lower *n* at short wavelengths (*n* (500 nm) = 2.278) and a pronounced growth toward the near-IR (*n* (2500 nm) = 4.572 and *n* (3000 nm) = 4.907), with an overall increase of +2.629 between 500 and 3000 nm. The strongest dispersion is observed for Ag/PANI—15%, where *n* rises from 3.563 (500 nm) to 9.599 (2500 nm) and 12.726 (3000 nm), with a peak *n* ≈ 12.829 around 2663 nm and strong oscillatory features in the 2600–2800 nm region. Such high-*n*, highly dispersive behavior at 15% is consistent with intense free-carrier/metal-related optical response and microstructure-dependent scattering in the strongly loaded nanocomposite. Overall, Figure 7 demonstrates that an increasing Ag concentration markedly enhances the extinction coefficient and increases the refractive index, shifting the system toward a more optically dense and lossy medium. The progressive rise in *K* and *n* especially for 15 wt.% supports stronger electronic polarization and broadband attenuation, plausibly driven by Ag-induced carrier density, interfacial charge transfer with the π-conjugated PANI backbone, and increased disorder/heterogeneity at higher nanoparticle loading [30].

- **Optical Anisotropy and Birefringence Analysis**

Optical anisotropy in the PANI and Ag/PANI films can be quantified using the dispersion of the refractive index, *n*(*λ*), extracted from Figure 7b. For each specimen, the ordinary index (*n*_0_) was approximated by the minimum *n*(*λ*) within the measured window (≈500–3000 nm), while the extraordinary index (*n_e_*) was approximated by the maximum *n*(*λ*) in the same range. The birefringence was then evaluated as Δ*n* = *n_e_ − n*_0_, and a uniaxial average refractive index was estimated using *n̄* = (2*n*_0_ *+ n_e_*)/3. This approach provides a consistent, figure-based measure of the magnitude of optical anisotropy and its dependence on Ag loading. Pristine PANI exhibits a weakly dispersive refractive index with *n*_0_ = 2.070 and *n_e_* = 2.545, giving a small birefringence Δ*n* = 0.475 and an average index *n̄* = 2.228. The low Δ*n* indicates limited structural anisotropy and a relatively homogeneous polymer matrix, where the optical response is dominated by the π-conjugated backbone with modest long-wavelength dispersion. Introducing Ag NPs amplifies anisotropy substantially. For Ag/PANI—5 wt.%, the refractive index spans from *n*_0_ = 3.603 to *n_e_* = 8.194, yielding Δ*n* = 4.591 and *n̄* = 5.133. Relative to pristine PANI, Δn increases by 4.116 (a factor of 9.66×), while the average index rises by +2.905 (from 2.228 to 5.133, +130.4%). The large index spread and the pronounced *n* maximum indicate strong optical polarizability and enhanced dispersion, consistent with significant interfacial polarization and microstructural anisotropy generated by the metallic nanophase within the PANI host. For Ag/PANI—10 wt.%, the refractive index range is *n*_0_ = 1.932 to *n_e_* = 4.907, corresponding to Δ*n* = 2.975 and *n̄* = 2.924. Compared with PANI, Δ*n* increases by 2.500 (6.26×), and *n̄* increases by +0.696 (+31.2%). Notably, the presence of the lowest *n*_0_ value (1.932) together with a moderate *n_e_* suggests that, at 10 wt.% loading, the optical response reflects a balance between increased carrier-related dispersion and microstructure-dependent effects (e.g., porosity, percolation, or nanoparticle distribution) that can reduce the effective index at certain wavelengths. The strongest optical anisotropy is observed for Ag/PANI—15 wt.%, where *n*_0_ = 3.419 and *n_e_* = 12.829, giving an exceptionally large birefringence Δ*n* = 9.410 and *n̄* = 6.556. Relative to PANI, Δ*n* increases by 8.935 (19.81×) and *n̄* increases by +4.328 (+194.3%).

The very high *n_e_* and large Δ*n* are consistent with a highly dispersive, optically dense response dominated by Ag-related free-carrier/metallic polarization and strong interfacial charge-transfer effects. The pronounced oscillations in *n*(*λ*) reported for the high-loading film in the 2600–2800 nm region further support the presence of strong wavelength-dependent dispersion driven by microstructure and increased optical loss mechanisms in the nanocomposite. Overall, the birefringence evolves nonlinearly with Ag concentration: Δ*n* increases from 0.475 (PANI) to 4.591 (5 wt.%), decreases to 2.975 (10 wt.%), and then rises sharply to 9.410 (15 wt.%). This non-monotonic progression indicates that anisotropy is governed not only by nanoparticle fraction but also by dispersion state and mesoscale morphology. Moderate Ag NP loading can generate strong interfacial polarization and anisotropic domains (large Δ*n* at 5 wt.%), whereas further loading may alter packing/porosity or percolation pathways (lower Δ*n* at 10 wt.%), before ultimately transitioning to a strongly metal-influenced optical regime at 15 wt.%, where polarization and dispersion dominate (Δ*n* = 9.410). These results confirm that Ag NP incorporation significantly enhances optical anisotropy and birefringence in PANI-based films, which is relevant for photonic components requiring tunable refractive indices and polarization-dependent optical response (Table 2).

- **The optical conductivity**

**Figure 8:**
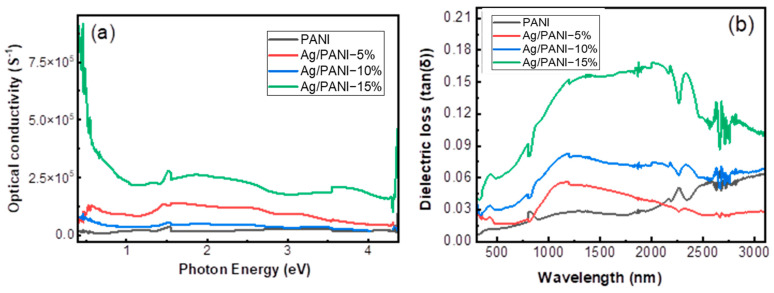
Representation of (**a**) optical conductivity and (**b**) dielectric loss (*tan*(*δ*)) for PANI and nanocomposites.

Figure 8a depicts the spectral variation in the optical conductivity, *σ_opt_* (S^−1^), for pristine PANI and Ag/PANI nanocomposite as a function of photon energy (*hν*). The optical conductivity was evaluated from the absorption coefficient (*α*) and refractive index (*n*) according to

(7) $$\sigma_{opt}=\frac{\alpha nc}{4\pi }$$

where is the speed of light in a vacuum. Because *σ_opt_* scales linearly with both *α* and *n*, any Ag-induced enhancement in optical loss (*α*) and optical density (*n*) is expected to translate directly into higher *σ_opt_*, especially in the low-energy region where free-carrier and polaronic contributions dominate. The spectra show a pronounced, loading-dependent increase in σ_opt over the entire photon-energy window. At *hν* = 0.50 eV, *σ_opt_* increases from 1.32 × 10^4^ S^−1^ for PANI to 1.24 × 10^5^ S^−1^ for Ag/PANI—5%, 5.70 × 10^4^ S^−1^ for Ag/PANI—10%, and 3.71 × 10^5^ S^−1^ for Ag/PANI—15%. Relative to PANI at the same energy, these correspond to enhancement factors of 9.42× (5%), 4.33× (10%), and 28.19× (15%). This large amplification at low *hν* is consistent with the strong increase in long-wavelength extinction and refractive index reported for the higher-loading nanocomposites. In the intermediate-energy region, *σ_opt_* remains clearly separated by composition and exhibits a broad, slowly varying response. At *hν* = 2.00 eV, *σ_opt_* equals 2.12 × 10^4^ S^−1^ (PANI), 1.26 × 10^5^ S^−1^ (5%), 5.01 × 10^4^ S^−1^ (10%), and 2.56 × 10^5^ S^−1^ (15%), meaning that the 15 wt.% film sustains a 12.03× higher *σ_opt_* than pristine PANI. At *hν* = 3.00 eV, *σ_opt_* is 2.93 × 10^4^ S^−1^ (PANI), 9.51 × 10^4^ S^−1^ (5%), 3.74 × 10^4^ S^−1^ (10%), and 1.81 × 10^5^ S^−1^ (15%). Thus, the 15 wt.% film maintains a 6.16× enhancement relative to PANI while the 5 wt.% film remains 3.24× higher than PANI. A key spectral feature is the strong low-energy conductivity of the 15 wt.% nanocomposite.

The extracted maximum optical conductivity for Ag/PANI—15% reaches 8.23 × 10^5^ S^−1^ at the lowest-energy edge of the plot and then decreases to ~2.21 × 10^5^ S^−1^ by 1.00 eV. This behavior is characteristic of a free-carrier-dominated optical response, where *σ_opt_* is highest at low photon energies and diminishes as the excitation energy moves away from the carrier-related absorption tail. In contrast, Ag/PANI—5% exhibits its highest *σ_opt_* in the mid-energy region, peaking at 1.44 × 10^5^ S^−1^ around *hν* ≈ 1.48 eV, then gradually declining toward higher energies. At the higher-energy end (*hν* ≈ 4.00 eV), *σ_opt_* remains elevated for the Ag-rich film: 1.77 × 10^5^ S^−1^ (15%) compared with 2.47 × 10^4^ S^−1^ (PANI), corresponding to a 7.17× enhancement. Minor fluctuations and edge spikes near the plot boundaries are attributed to reduced signal stability and numerical sensitivity in *α* and *n* extraction at the spectral limits, and therefore the central-energy trends provide the most reliable comparison. Overall, Figure 8a demonstrates that Ag NP incorporation drives a substantial, composition-controlled increase in optical conductivity across the measured photon energies. The hierarchy Ag/PANI—15% ≫ Ag/PANI—5% > Ag/PANI—10% > PANI is consistent with the simultaneous Ag-induced enhancement of extinction (*α*) and refractive index (*n*). The large low-energy *σ_opt_* of the 15 wt.% film supports a transition toward a more metal-influenced, carrier-rich optical response, likely arising from intensified interfacial charge transfer, higher free-carrier density, and stronger electronic delocalization in the Ag/PANI network [34].

### 3.5. Dielectric Properties

#### 3.5.1. Wavelength-Dependent Dielectric Response

**Figure 9:**
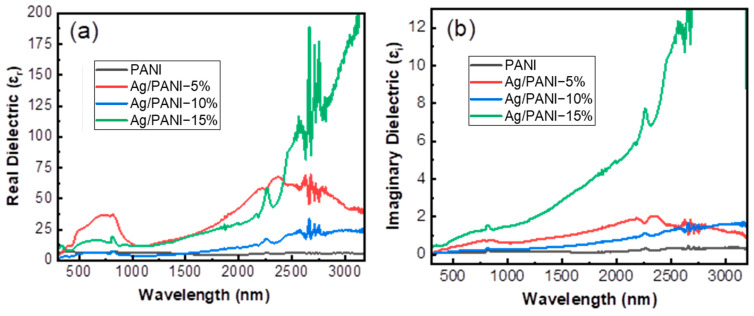
(**a**) Real dielectric (*ε_r_*) and (**b**) imaginary dielectric (*ε_i_*) for PANI and nanocomposites.

Figure 9 resolves the wavelength-dependent complex dielectric response of PANI and Ag/PANI nanocomposite into the real part, *ε_r_*, and the imaginary part, *ε_i_*, over the broad near-UV/visible to near-IR window (≈400–3200 nm). The dielectric function is expressed as a complex quantity:

(8) $$\varepsilon \;=\varepsilon_{r}\;+\;{i\varepsilon }_{i}$$

where *ε_r_* describes the ability of the medium to store electromagnetic energy through polarization, while *ε_i_* represents dielectric loss associated with energy dissipation. Importantly, *ε_r_* and *ε_i_* can be derived directly from the optical constants (refractive index *n* and extinction coefficient *k*) through

(9) $$\varepsilon_{r}=n^{2\;}-k^{2}$$

(10) $${\;\;\;\varepsilon }_{i}=2nk$$

Thus, increases in n primarily elevate *ε_r_*, whereas increases in *k* (optical loss) strongly amplify *ε_i_*. This linkage is especially relevant here because Ag NP loading simultaneously modifies both *n* and *k*, thereby reshaping polarization and loss. Pristine PANI exhibits a low-magnitude and weakly dispersive *ε_r_* throughout most of the spectrum, indicating modest polarizability and limited long-wavelength dispersion. Quantitatively, *ε_r_* (PANI) is 4.110 at 500 nm and remains 4.110 at 1000 and 1500 nm, before dropping to 2.740 at 2000 nm and recovering slightly to 3.196 at 2500–3000 nm. The narrow range (2.740–4.110) reflects a relatively stable electronic polarization response, consistent with a polymer matrix where dispersion is governed mainly by π–π* transitions at higher energies and polaronic contributions at lower energies without a strong metallic component. Introducing Ag nanoparticles substantially increases *ε_r_* and makes it strongly wavelength-dependent, evidencing enhanced electronic and interfacial polarization. For Ag/PANI—5 wt.%, *ε_r_* rises from 8.676 (500 nm) to 13.699 (1000 nm) and 14.612 (1500 nm), then accelerates to 35.160 at 2000 nm and reaches a maximum of 58.904 at 2500 nm before decreasing to 48.402 at 3000 nm. Relative to PANI, the enhancement factors at 2000 nm and 2500 nm are 12.83× (35.160/2.740) and 18.43× (58.904/3.196), respectively. This sharp long-wavelength growth indicates that Ag incorporation increases the effective polarizability, likely through a (**i**) higher carrier density, (**ii**) Maxwell–Wagner-type interfacial polarization at Ag/PANI boundaries, and (**iii**) stronger dispersion as the system approaches a more conductive regime. The Ag/PANI—10 wt.% sample shows a distinct profile: *ε_r_* is comparatively low at short wavelengths (0.457 at 500 nm, 1.370 at 1000 nm) but increases steadily toward the near-IR (2.283 at 1500 nm, 7.306 at 2000 nm, 15.525 at 2500 nm, and 21.461 at 3000 nm). The growth from 500 to 3000 nm equals +21.004, indicating pronounced long-wavelength dispersion even though the absolute *ε_r_* values are smaller than those of 5% at 2000–2500 nm. This behavior implies that the effective *n*^2^ term (Equation (9)) increases progressively with the wavelength and that microstructural factors (e.g., porosity, percolation topology, or nanoparticle distribution) can reduce the effective *ε_r_* in the visible while still enabling strong near-IR polarization. The strongest *ε_r_* enhancement is observed for Ag/PANI—15 wt.%, which transitions to a highly dispersive dielectric response at long wavelengths. *ε_r_* increases from 5.479 (500 nm) to 9.589 (1000 nm) and 13.242 (1500 nm), then rises to 25.114 at 2000 nm, 61.187 at 2500 nm, and reaches 146.119 at 3000 nm. At 3000 nm, *ε_r_* (15%) exceeds *ε_r_* (PANI) by 45.71 × (146.119/3.196), evidencing a dramatic increase in optical polarizability and a strong low-energy response consistent with metal-assisted dispersion and carrier-rich behavior. The sharp increase and oscillatory features near the far-right spectral edge suggest that extraction becomes sensitive to measurement noise and to the strong loss regime; nevertheless, the magnitude and trend clearly demonstrate that a high Ag content drives *ε_r_* to very large values in the near-IR.

Pristine PANI exhibits very low *ε_i_* values, consistent with weak optical loss. *ε_i_* (PANI) equals 0.237 at 500 nm, remains 0.297 at 1000–2000 nm, and then increases slightly to 0.416 at 2500 nm and 0.505 at 3000 nm. The modest long-wavelength rise indicates increasing absorption/loss at low photon energies, but the absolute values remain small, confirming that pristine PANI is comparatively low-loss in this window. Ag NP incorporation increases *ε_i_* markedly and, in most cases, shifts loss toward longer wavelengths. For Ag/PANI—5 wt.%, *ε_i_* rises from 0.386 at 500 nm to 0.772 at 1000 nm, 1.039 at 1500 nm, and 1.632 at 2000 nm, reaching 1.692 at 2500 nm before slightly decreasing to 1.543 at 3000 nm. Relative to PANI, *ε_i_* is enhanced by 5.49× at 2000 nm (1.632/0.297) and by 4.07× at 2500 nm (1.692/0.416). This evolution indicates that *k* increases strongly with Ag NP loading and that absorption-related loss becomes significant in the near-IR, consistent with free-carrier absorption and interfacial dissipation. The Ag/PANI—10 wt.% film shows a more gradual increase in *ε_i_*, from 0.237 at 500 nm to 0.416 at 1000 nm, 0.534 at 1500 nm, 0.861 at 2000 nm, 1.336 at 2500 nm, and 1.692 at 3000 nm. At 3000 nm, *ε_i_* (10%) is 3.35× higher than *ε_i_* (PANI) (1.692/0.505). The monotonic increase suggests a steadily growing loss contribution with wavelength, matching the observed increase in optical loss (k) at long wavelengths for this loading. The highest dielectric loss is achieved for Ag/PANI—15 wt.%. *ε_i_* rises from 0.623 at 500 nm to 1.514 at 1000 nm, 2.582 at 1500 nm, 4.600 at 2000 nm, and 8.785 at 2500 nm, reaching 12.436 at 2700 nm. At 2000 nm, *ε_i_* (15%) is 15.49× larger than PANI (4.600/0.297), demonstrating that Ag-rich films are strongly dissipative at low photon energies. At the far-right edge (≈2700–3000 nm), the *ε_i_* curve approaches the plotted upper limit and shows oscillations; therefore, *ε_i_* at 3000 nm is treated as not reliable for robust digitization.

This limitation is expected when k becomes large and the derivation of *ε_i_* (Equation (10)) becomes highly sensitive to spectral noise and to the exact *n–k* extraction. Equations (9) and (10) rationalize why the Ag/PANI—15 wt.% film simultaneously exhibits very large *ε_r_* and *ε_i_* at long wavelengths: *n* rises strongly in the near-IR and *k* also increases, so *ε_r_* (≈*n*^2^) is boosted while *ε_i_* (≈2*nk*) grows even more rapidly. In contrast, the 5 wt.% film displays high *ε_r_* and *ε_i_* in the 2000–2500 nm region but slightly lower values at 3000 nm, consistent with a scenario where dispersion and loss peak in mid-IR/near-IR due to the particular nanoparticle distribution and carrier relaxation landscape. The 10 wt.% film shows a more moderate *ε_i_* but a steadily increasing *ε_r_* and *ε_i_* toward 3000 nm, suggesting a smoother evolution of *n* and *k* with the wavelength. Overall, Figure 9 demonstrates that Ag incorporation transforms the optical dielectric response of PANI from a low-loss, weakly dispersive polymer (*ε_r_* ≈ 2.740–4.110; *ε_i_* ≈ 0.237–0.505) into a strongly dispersive and increasingly lossy nanocomposite. The strongest transformation occurs at 15 wt.% Ag, where *ε_r_* reaches 146.119 at 3000 nm and *ε_i_* reaches 12.436 at 2700 nm, indicating a metal-influenced, carrier-rich regime dominated by enhanced polarization and dissipation. These quantitative trends are consistent with intensified interfacial charge transfer, increased free-carrier density, and strengthened Maxwell–Wagner polarization losses as the Ag NP fraction increases [35].

#### 3.5.2. The Dielectric Loss Tangent (*tan δ*)

Figure 8b shows the spectral evolution of the dielectric loss tangent (*tan δ*) (Equation (11)) for pristine PANI and Ag/PANI nanocomposites as a function of wavelength (≈400–3200 nm). The loss tangent is a dimensionless measure of the balance between dissipated and stored electromagnetic energy in the medium and is defined by

(11) $$\tan\left(\delta \right)=\frac{\varepsilon_{i}}{\varepsilon_{r}}$$

where *ε_r_* is the real dielectric component associated with energy storage (polarization) and *ε_i_* is the imaginary component associated with dielectric loss (dissipation). Therefore, *tan*(*δ*) increases when loss processes (*ε_i_*) grow faster than the polarization term (*ε_r_*), and decreases when *ε_r_* dominates. Across the entire wavelength window, *tan*(*δ*) increases markedly with Ag NP loading, demonstrating that Ag NP incorporation introduces additional dissipation channels (e.g., interfacial polarization relaxation and carrier-related absorption) in the PANI matrix. Pristine PANI (black) exhibits the lowest loss level: *tan*(*δ*) equals 0.058 at 500 nm and increases slightly to 0.072 at 1000 nm and 0.072 at 1500 nm. In the near-IR, the loss rises further as the absorption tail becomes more important, giving *tan*(*δ*) = 0.108 at 2500 nm and 0.158 at 3000 nm. The overall increase from 500 to 3000 nm is +0.100, indicating that PANI becomes progressively more dissipative at long wavelengths, but the magnitude remains relatively modest. For Ag/PANI—5 wt.% (red), *tan*(*δ*) is higher than pristine PANI over most of the spectrum and shows a broad high-loss region in the near-IR/short-wave IR. Quantitatively, *tan*(*δ*) increases from 0.044 at 500 nm to 0.056 at 1000 nm and 0.071 at 1500 nm, then reaches 0.046 at 2000 nm and decreases to 0.029 at 2500 nm and 0.032 at 3000 nm. This behavior reflects the fact that *ε_r_* grows strongly for 5 wt.% (large polarization storage), which can partially suppress *tan*(*δ*) even when *ε_i_* increases [36]. In other words, the 5 wt.% composite becomes more polarizable (higher *ε_r_*) faster than it becomes lossy (*ε_i_*), lowering *ε_i_*/*ε_r_* at the long-wavelength side. Ag/PANI–10 wt.% (blue) exhibits a higher loss level than 5 wt.% over a wider portion of the spectrum. The calculated *tan*(*δ*) equals 0.519 at 500 nm, decreases to 0.304 at 1000 nm and 0.234 at 1500 nm, and then remains in the 0.118–0.079 range at longer wavelengths (0.118 at 2000 nm, 0.086 at 2500 nm, and 0.079 at 3000 nm). The comparatively large value at 500 nm originates from the combination of small *ε_r_* and finite *ε_i_* at short wavelengths for this composition, highlighting the sensitivity of *tan*(*δ*) to the relative magnitudes of *ε_i_* and *ε_r_*. The highest dielectric loss is obtained for Ag/PANI—15 wt.% (green). *tan*(*δ*) equals 0.114 at 500 nm, 0.158 at 1000 nm, 0.195 at 1500 nm, 0.183 at 2000 nm, and 0.144 at 2500 nm. In the far near-IR (≈2700–3000 nm), the curve approaches the upper plotting limit and displays oscillatory behavior; therefore, *tan*(*δ*) at 3000 nm is not treated as a robust digitized value. Nevertheless, the consistently larger *tan*(*δ*) values for the 15 wt.% film indicate that dissipation grows strongly with Ag loading, consistent with enhanced *k*-driven losses and a more metal-influenced optical response. Overall, the *tan*(*δ*) hierarchy across most wavelengths is Ag/PANI—15% > Ag/PANI—10% > Ag/PANI—5% > PANI, confirming that Ag nanoparticles promote dielectric loss. Because *tan*(*δ*) is defined by *ε_i_*/*ε_r_* (Equation (11)), the observed trends reflect the coupled evolution of *ε_r_* and *ε_i_*: Ag NP loading increases *ε_i_* through stronger absorption and carrier-related dissipation, while also increasing *ε_r_* through enhanced polarization. The net *tan*(*δ*) response is governed by which term grows faster at a given wavelength, explaining why compositions with very large *ε_r_* can show moderate *tan*(*δ*) even when *ε_i_* is elevated [37,38,39,40,41].

**Figure 10:**
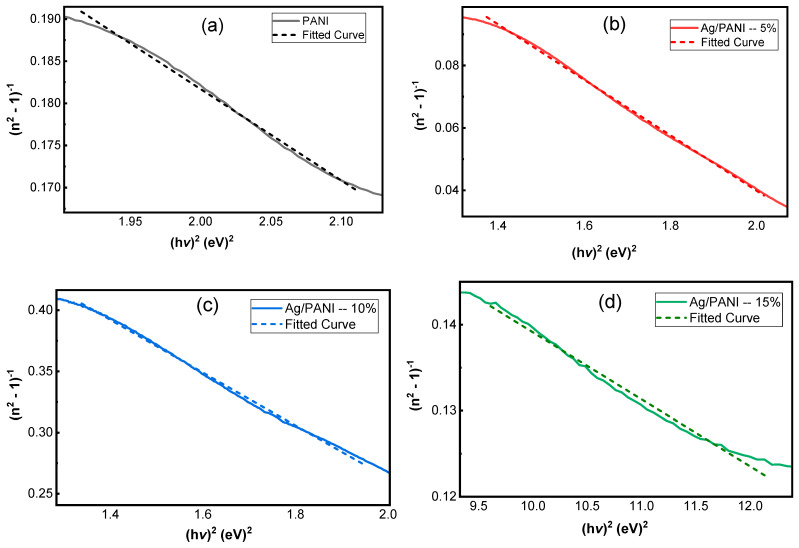
Plot of (*n*^2^ − 1)^−1^ versus photon energy squared ((*hν*)^2^) for (**a**) PANI and Ag/PANI nanocomposites: (**b**) 5%, (**c**) 10%, and (**d**) 15% Ag. Experimental data and fitted lines demonstrate linear dispersion and Ag-induced modification of optical parameters.

Figure 10a–d presents the Wemple–DiDomenico (single-oscillator) analysis of the dispersion of the refractive index for pristine PANI and Ag/PANI nanocomposites through the linear representation of ${\left(n^{2}\;-\;1\right)}^{-1}$ as a function of ${\left(h\nu \right)}^{2}$. In this model, the refractive-index dispersion in the transparent to weakly absorbing region is described by

(12) $${\left(n^{2}-1\right)}^{-1}=\frac{E_{0}}{E_{d}}-\left(\frac{1}{E_{0}E_{d}}\right){\left(h\nu \right)}^{2}$$

where *E*_0_ is the single-oscillator energy and *E_d_* is the dispersion energy, which is proportional to the strength of interband optical transitions. Equation (12) predicts a linear dependence of ${\left(n^{2}\;-\;1\right)}^{-1}$ on ${\left(h\nu \right)}^{2}$ with an intercept of *E*_0_/*E_d_* and a negative slope of −1/(*E*_0_*E_d_*). Accordingly, the quality of the linear fit in Figure 10 indicates that the single-oscillator approximation describes the measured dispersion in the selected spectral ranges. The extracted parameters (Table 3) further yield the zero-frequency refractive index and static dielectric constant via

(13) $$n_{0}=\sqrt{1+\frac{E_{0}}{E_{d}}}$$

(14) $$\varepsilon_{s}=n_{0}^{2}$$

together with additional derived quantities frequently used for comparative dispersion analysis [42,43,44]:

(15) $$f\;=\;E_{0}E_{d}$$

(16) $$M_{-1}=\frac{E_{d}}{E_{0}}$$

(17) $$M_{-3}=\frac{E_{d}}{E_{0}^{3}}$$

where *f* is the oscillator strength and *M*_−1_, *M*_−3_ are spectral moments. A key outcome of Figure 10 is that all samples exhibit an approximately linear decrease of ${\left(n^{2}\;-\;1\right)}^{-1}$ with increasing ${\left(h\nu \right)}^{2}$, consistent with the negative slope required by Equation (12). The magnitude of the slope is controlled by −1/(*E*_0_*E_d_*) and therefore tracks the oscillator strength *f* (Equation (15)): a larger *f* yields a smaller-magnitude slope (less steep line), while a smaller f yields a steeper line. Using the Table 3 values, the slopes are −0.1083 eV^−2^ for PANI, −0.0892 eV^−2^ for Ag/PANI—5 wt.%, −0.2165 eV^−2^ for Ag/PANI—10 wt.%, and −0.0078 eV^−2^ for Ag/PANI—15 wt.%. The 15 wt.% film therefore displays the least steep dispersion line (|slope| reduced by 13.88× relative to PANI), reflecting its very large *f* = 128.21 eV^−2^. In contrast, the 10 wt.% film has the steepest line (|slope| = 0.2165 eV^−2^), consistent with the smallest oscillator strength (*f* = 4.63 eV^−2^) among the nanocomposites. The intercept term *E*_0_/*E_d_* also differs among the compositions and provides direct access to *n*_0_ through Equation (13). From Table 3, PANI shows *E*_0_ = 1.92 eV and *E_d_* = 4.81 eV, giving an intercept *E*_0_/*E_d_* = 0.3992 and *n*_0_ = 1.1829 (*ε_s_* = 1.3992). Ag incorporation modifies both *E*_0_ and *E_d_*. For Ag/PANI—5 wt.%, *E*_0_ = 1.56 eV and *E_d_* = 7.19 eV, yielding *E*_0_/*E_d_* = 0.2170 and *n*_0_ = 1.1032 (*ε_s_* = 1.2170). This indicates that although *E_d_* increases significantly relative to pristine PANI (+49.48%), the ratio *E*_0_/*E_d_* decreases, leading to a reduced *n*_0_ (−6.75% relative to PANI). For Ag/PANI—10 wt.%, *E*_0_ = 1.79 eV and *E_d_* = 2.58 eV, giving the largest intercept *E*_0_/*E_d_* = 0.6938 and thus the largest *n*_0_ = 1.3015 (*ε_s_* = 1.6938). This composition therefore exhibits the highest predicted static dielectric response among the reliable WDD fits, with *ε_s_* increasing by +0.2946 relative to PANI (+21.05%). For Ag/PANI—15 wt.%, the parameters shift dramatically to *E*_0_ = 5.27 eV and *E_d_* = 24.30 eV, yet the ratio *E*_0_/*E_d_* = 0.2169 remains close to the 5 wt.% value, producing *n*_0_ = 1.1031 and *ε_s_* = 1.2169. Thus, the 15 wt.% sample combines an extremely large *E_d_* with a much larger *E*_0_, preserving a small *E*_0_/*E_d_* ratio and hence a modest *n*_0_. The dispersion energy *E_d_* is particularly informative because it reflects the average strength of interband transitions and is sensitive to bonding, coordination, and microstructure. Relative to PANI (*E_d_* = 4.81 eV), the 5 wt.% film increases to 7.19 eV, suggesting a strengthened effective transition strength, plausibly due to enhanced electronic coupling and interfacial polarization at Ag-PANI boundaries. The 10 wt.% film decreases to 2.58 eV, indicating weaker average dispersion strength in the fitted region, which can arise from changes in morphology (e.g., porosity, dispersion state) or the redistribution of oscillator strength into loss channels captured by *k* rather than by the purely dispersive n term. The 15 wt.% film shows a very high *E_d_* = 24.30 eV, consistent with a strong overall optical transition strength typical of a highly polarizable, metal-influenced composite; however, this is accompanied by a high *E*_0_ = 5.27 eV, indicating that the effective oscillator energy shifts to higher energies.

The spectral moments derived further clarify these trends. M_−1_ = *E*_0_/*E_d_* increases from 2.5052 eV^−2^ (PANI) to 4.6090 eV^−2^ (5 wt.%) and 4.6110 eV^−2^ (15 wt.%), while it drops to 1.4413 eV^−2^ at 10 wt.%. Similarly, M_−3_ increases strongly at 5 wt.% (1.8939 eV^−2^) but becomes very small at 15 wt.% (0.1660 eV^−2^) because of the cubic dependence on *E*_0_ (Equation (17)). This demonstrates that the 15 wt.% sample’s high *E*_0_ suppresses *M_−_*_3_ even when *E_d_* is very large. The oscillator strength *f* = *E*_0_*E_d_* follows the order 15 wt.% (128.21) ≫ 5 wt.% (11.25) > PANI (9.23) > 10 wt.% (4.63), which is consistent with the relative steepness of the lines in Figure 10 via the slope term in Equation (12). Overall, Figure 10 and Table 3 show that Ag NP loading does not simply increase or decrease all dispersion parameters monotonically; rather, it reshapes the balance between oscillator energy (*E*_0_) and dispersion energy (*E_d_*). Moderate loading (5 wt.%) strengthens dispersion energy and oscillator strength but reduces *n*_0_ and *ε_s_* due to a smaller *E*_0_/*E_d_* ratio, whereas 10 wt.% yields the highest *n*_0_ and *ε_s_* because it maximizes *E*_0_/*E_d_*. High loading (15 wt.%) produces extremely large *E_d_* and f with a shallow dispersion slope, consistent with a strongly polarizable composite, but retains a modest *n*_0_ because *E*_0_ increases concurrently. These quantitative results support the conclusion that the optical dispersion in Ag/PANI films is governed by a coupled evolution of transition strength, effective oscillator energy, and microstructure-dependent electronic coupling.

#### 3.5.3. Nonlinear Optical Parameters

Table 4 summarizes the linear susceptibility ($\chi^{1}$), the third-order nonlinear susceptibility ($\chi^{3}$), and the nonlinear refractive index ($n_{2}$) for pristine PANI and Ag/PANI nanocomposites. These parameters quantify the strength of light–matter interaction in the linear and third-order nonlinear regimes and are central for evaluating the suitability of the films for nonlinear photonic applications (optical switching, limiting, and all-optical modulation). The following relations, given in Equations (18)–(22), were used to evaluate the nonlinear optical parameters from the experimentally derived optical constants. The macroscopic polarization *P* induced by an applied optical electric field *E* can be expanded as

(18) $$P=\;\chi^{1}E+\chi^{2}E^{2}+\chi^{3}E^{3}$$

where $\chi^{1}$ is the linear susceptibility, $\chi^{2}$ is the second-order susceptibility (often suppressed in centrosymmetric media), and $\chi^{3}$ is the third-order susceptibility that governs Kerr nonlinearity and nonlinear absorption [45]. Within the Wemple–DiDomenico-based formalism used here, $\chi^{1}\;$ and $\chi^{3}$ can be related to the dispersion parameters ($E_{d}$ and $E_{0}$) [46,47,48] by

(19) $$\chi^{1}=\frac{E_{d}}{4\pi E_{0}}$$

(20) $$\chi^{3}=1.7\times 10^{-10}\times {\left(\frac{E_{d}}{E_{0}}\right)}^{4}\;\;\;\;\;\;(e.s.u.)$$

The optical refractive index can similarly be written as a field-dependent expansion:

(21) $$n(E)\;=\;n_{0}\;+\;n_{1}E+\;n_{2}E^{2}+\;\ldots ,\;$$

and the nonlinear refractive index $n_{2}\;$ is linked to $\chi^{3}$ [49,50] through

(22) $$n_{2}=\frac{12\pi \chi^{3}}{n_{0}}$$

highlighting that a larger $\chi^{3}$ (and smaller $n_{0}$) generally promotes stronger Kerr nonlinearity. In these equations, each parameter retains its usual optical meaning, and the set of relations was applied to obtain the composition-dependent nonlinear parameters of the Ag/PANI films. The observed U-shaped dependence of the dispersion parameters and Kerr nonlinearity suggests that the optical response is governed by a balance between Ag-induced enhancement and microstructural disruption, rather than by the filler concentration alone. At low Ag loading, an improved nanoparticle dispersion and strong Ag/PANI interfacial coupling can enhance local electromagnetic fields, interfacial polarization, and charge-transfer-assisted polarizability, leading to an increased dispersion strength and nonlinear response. At the intermediate 10 wt.% composition, partial nanoparticle aggregation and microstructural reorganization may weaken these cooperative effects by disrupting polarization continuity and reducing the efficiency of local-field enhancement, thereby producing the observed dip. Upon a further increase to 15 wt.% Ag, the larger metallic contribution appears to restore the overall polarizability and carrier-related optical response, resulting in renewed enhancement of the dispersion and Kerr-related parameters. Therefore, the non-monotonic trend reflects a competition between interfacial/local-field amplification and disorder-induced disruption within the nanocomposite structure. Pristine PANI shows $\chi^{1}$ = 0.2. Incorporating Ag at 5 wt.% increases $\chi^{1}$ to 0.4, i.e., a +0.2 absolute increment corresponding to a 2.00× enhancement relative to PANI. A similar $\chi^{1}$ = 0.4 is obtained for the 15 wt.% nanocomposite, again 2.00× higher than pristine PANI. In contrast, Ag/PANI—10 wt.% exhibits $\chi^{1}$ = 0.1, which is half the PANI value (0.50×) and indicates a reduction in the effective linear polarizability at this intermediate composition. This non-monotonic behavior suggests that $\chi^{1}$ is governed not only by the Ag NP fraction but also by microstructural factors that influence the effective oscillator strength and local field distribution. The most striking effect of Ag loading is the strong amplification of $\chi^{3}$ at 5 wt.% and 15 wt.%. Pristine PANI exhibits $\chi^{3}$ = 6.73 × 10^−9^ e.s.u. Ag/PANI—5 wt.% increases to $\chi^{3}$ = 75.82 × 10^−9^ e.s.u., which is an absolute rise of +69.09 × 10^−9^ e.s.u. and an enhancement factor of 11.27× relative to PANI. Ag/PANI—15 wt.% yields $\chi^{3}$ = 76.56 × 10^−9^ e.s.u., corresponding to +69.83 × 10^−9^ e.s.u. compared with PANI and a factor of 11.38× enhancement. The 5 wt.% and 15 wt.% values are also very close to each other, differing by only 0.74 × 10^−9^ e.s.u. (≈0.98%), indicating that the system reaches a near-saturation level of $\chi^{3}$ once a sufficiently strong Ag-induced electronic response and interfacial polarization are established. Conversely, Ag/PANI—10 wt.% shows a very low $\chi^{3}\;$= 0.73 × 10^−9^ e.s.u., which is 9.22× lower than pristine PANI and 103.9× lower than the 5 wt.% nanocomposite. Such suppression at 10 wt.% indicates that the nonlinear response is extremely sensitive to the balance between dispersion energy, oscillator energy, and local-field enhancement; microstructural redistribution (e.g., aggregation/percolation state, disorder, or void fraction) can strongly diminish the effective third-order polarization even when the metal content is increased. The $n_{2}$ values follow the same qualitative pattern as $\chi^{3}$, but with composition-dependent scaling. PANI shows $n_{2}$ = 2.15 × 10^−7^ e.s.u. A dramatic enhancement is observed for Ag/PANI—5 wt.% where $n_{2}$ = 25.91 × 10^−7^ e.s.u., i.e., +23.76 × 10^−7^ e.s.u. relative to PANI and a 12.05× increase. Ag/PANI—10 wt.% drops to $n_{2}$ = 0.21 × 10^−7^ e.s.u., which is 10.24× lower than PANI and 123.4× lower than the 5 wt.% nanocomposite. Interestingly, although Ag/PANI—15 wt.% maintains a very high $\chi^{3}$ (76.56 × 10^−9^ e.s.u.), the reported *n*_2_ is only 0.26 × 10^−7^ e.s.u., which is 8.27× lower than PANI and 99.7× lower than Ag/PANI—5 wt.%. This divergence between $\chi^{3}\;$and $n_{2}$ implies that the effective refractive Kerr coefficient at 15 wt.% is suppressed by additional factors embedded in Equation (22), most plausibly a large effective $n_{0\;}$ and/or strong competing loss/dispersion that alters the real part of the nonlinear index. In highly loaded nanocomposites, intense absorption and strong dispersion can reduce the measurable Kerr refraction and shift the nonlinear response toward absorptive contributions, even when $\chi^{3}$ remains large. Overall, Table 4 demonstrates that Ag incorporation can raise $\chi^{1}$ up to 0.4 and boost $\chi^{3}$ to ~76 × 10^−9^ e.s.u., establishing strong third-order nonlinearity in the 5 wt.% and 15 wt.% nanocomposites. Among the studied compositions, Ag/PANI—5 wt.% provides the most favorable combination of a high $\chi^{3}$ (75.82 × 10^−9^ e.s.u.) and very large $n_{2}$ (25.91 × 10^−7^ e.s.u.), making it the best candidate for Kerr-based nonlinear optical functionality. Because the optical constants were derived from the measured spectral response of heterogeneous films, the extracted refractive-index dispersion represents an effective optical behavior that may include contributions from morphology-related scattering in addition to true absorption. This effect is expected to be more pronounced at higher Ag loading, where AFM/SEM reveals increased roughness and aggregation; therefore, the derived dispersion and nonlinear parameters should be interpreted as effective nanocomposite optical constants rather than strictly scattering-free intrinsic values. The pronounced non-monotonic response at 10 wt.% and the $\chi^{3}$|$n_{2}$ decoupling at 15 wt.% underscore that the nonlinear optical performance is controlled by both the composition and microstructure, including the nanoparticle dispersion state, interfacial density, and local-field factors, rather than by the filler fraction alone.

**Figure 11:**
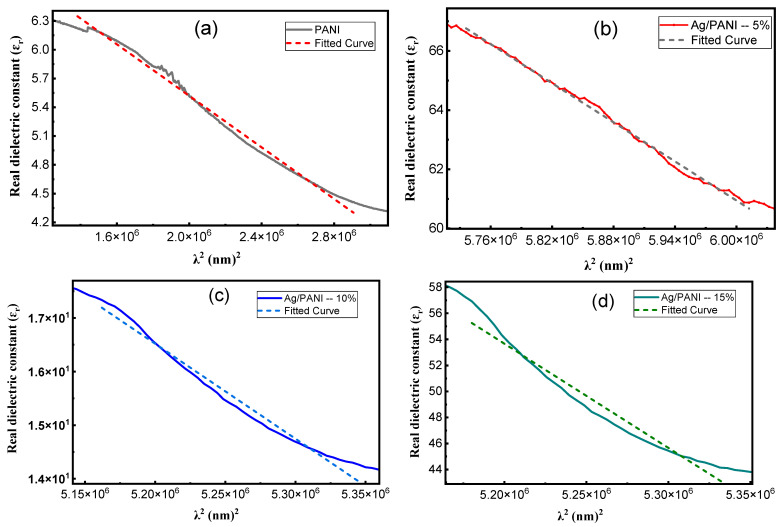
*ε_r_* versus *λ*^2^. Real dielectric constant (*εᵣ*) versus squared wavelength (*λ*^2^) for (**a**) PANI and Ag/PANI nanocomposites: (**b**) 5%, (**c**) 10%, and (**d**) 15% Ag. Solid curves show experimental data, and dashed curves represent fitted dispersion model results, revealing increased polarization with higher Ag loading.

Figure 11a–d plot the real part of the dielectric constant ($\varepsilon_{r}$) as a function of the squared wavelength ($\lambda^{2}$) for pristine PANI and Ag/PANI nanocomposites. The $\varepsilon_{r}\;$(*λ*) values were obtained from the optical constants *n* and *k* through $\varepsilon_{r}\;=\;n^{2\;}-\;k^{2}$, and the $\varepsilon_{r}$ versus $\lambda^{2}$ representation is interpreted using the Drude-type (Spitzer–Fan) free-carrier dispersion in the infrared. In this framework, $\varepsilon_{r}\;$ varies linearly with $\lambda^{2}$ as

(23) $$\varepsilon_{r}=n^{2\;}-k^{2}=\varepsilon_{l}-\left(\frac{e^{2}}{4\;\pi^{2}c^{2}\varepsilon_{0}}\right)\;\left(\frac{N}{m^{*}}\right)\;\lambda^{2}$$

where $\varepsilon_{l}\;$ is the high-frequency (lattice/ionic + bound-electron) dielectric constant, *e* is the elementary charge, *c* is the speed of light, $\varepsilon_{0}$ is the permittivity of free space, and $\frac{N}{m^{*}}$ is the carrier concentration-to-effective-mass ratio [50,51]. Here, the terms ‘free-carrier response’ and ‘carrier-related contribution’ are used in the optical sense, based on Drude-type fitting of the dielectric-dispersion behavior and the long-wavelength evolution of the optical constants, rather than on direct electrical measurements such as dc conductivity or Hall-effect analysis. Therefore, these interpretations should be understood as optically inferred trends consistent with enhanced carrier-related polarization/loss, not as direct experimental determination of carrier density. According to Equation (23), the $\varepsilon_{r}$ versus $\lambda^{2}$ plot should be approximately linear with (i) an intercept equal to $\varepsilon_{l}$ at $\lambda^{2}$ → 0 and (ii) a negative slope proportional to $\left(\frac{N}{m^{*}}\right)$. Thus, stronger free-carrier contributions (larger $\left(\frac{N}{m^{*}}\right))\;$ produce a steeper decrease in $\varepsilon_{r}\;$ with $\lambda^{2}$. Consistent with Equation (23), all panels in Figure 11 show a monotonic reduction in $\varepsilon_{r}$ with increasing $\lambda^{2}$, confirming that long-wavelength dispersion is governed by carrier-related polarization rather than by a purely bound-electron response. For pristine PANI (Figure 11a), $\varepsilon_{r}$ decreases smoothly across the shown $\lambda^{2}\;$ window, reflecting a weak but measurable carrier contribution. Table 5 quantifies the corresponding parameters: PANI exhibits $\varepsilon_{l}\;$= 8.2 and $\frac{N}{m^{*}}\;$= 1.6 × 10^39^ (kg^−1^ m^−3^), together with a plasma frequency *ω_p_* = 4.8 × 10^12^ Hz. These values establish PANI as a low-carrier, weakly dispersive polymer in the present spectral range. Upon incorporating Ag, the $\varepsilon_{r}\;$ versus $\lambda^{2}$ trends become markedly stronger, indicating an enhanced carrier density and/or reduced effective mass in the composite. For Ag/PANI—5 wt.% (Figure 11b), $\varepsilon_{r}$ decreases almost linearly with $\lambda^{2}$ and the extracted bound-electron dielectric constant rises to $\varepsilon_{l}\;$= 193.1. Relative to PANI, this is a 23.549× increase (193.1/8.2). Simultaneously, $\frac{N}{m^{*}}$ increases to 27.0 × 10^39^ (kg^−1^ m^−3^), i.e., a 16.875× enhancement over PANI (27.0/1.6). The plasma frequency rises to *ω_p_* = 78.2 × 10^12^ Hz, which is 16.292× higher than PANI (78.2/4.8). The concurrent growth of $\varepsilon_{l}$, $\frac{N}{m^{*}}$, and *ω_p_* indicates that even modest Ag loading substantially strengthens both the bound-electron polarizability and free-carrier response. Ag/PANI—10 wt.% (Figure 11c) also shows a strong $\varepsilon_{r}\;$ versus $\lambda^{2}$ decrease, with parameters that remain far above pristine PANI but below the 5 wt.% and 15 wt.% extremes. Specifically, $\varepsilon_{l}\;$= 108.6 (13.244× PANI), $\frac{N}{m^{*}}\;$= 21.7 × 10^39^ (13.562× PANI), and *ω_p_* = 62.9 × 10^12^ Hz (13.104× PANI). These values demonstrate that Ag addition increases the carrier-related dispersion term in Equation (23) substantially, but the response is not strictly monotonic with loading when moving from 5 to 10 wt.%. This non-monotonicity suggests that the effective free-carrier contribution is sensitive to the microstructure, including the Ag dispersion state, percolation connectivity, and interfacial charge-transfer efficiency, rather than being determined solely by the nominal filler fraction. The most pronounced effect is observed for Ag/PANI—15 wt.% (Figure 11d). The $\varepsilon_{r}\;$ versus $\lambda^{2}$ plot exhibits the steepest overall decline, consistent with the largest extracted $\left(\frac{N}{m^{*}}\right)$ in Table 5. Quantitatively, $\varepsilon_{l}\;$ increases dramatically to 469.8, which is 57.293× higher than PANI. The $\left(\frac{N}{m^{*}}\right)$ ratio reaches 98.0 × 10^39^ (kg^−1^ m^−3^), corresponding to a 61.250× enhancement relative to PANI. The plasma frequency increases to *ω_p_* = 248 × 10^12^ Hz, i.e., 51.667× PANI. Because *ω_p_* scales with carrier density (and inversely with effective mass), the very large *ω_p_* for 15 wt.% supports a transition toward a carrier-rich, metal-influenced optical response in the near-IR, consistent with the strong increases in optical loss and dielectric loss observed in the complementary figures. From Equation (23), the slope magnitude is proportional to $\left(\frac{N}{m^{*}}\right)$. Therefore, comparing Table 5 provides a direct quantitative ranking of the expected slopes: |slope| (15%) > |slope| (5%) > |slope| (10%) ≫ |slope| (PANI), in agreement with the qualitative ordering of steepness in Figure 11. In addition, the substantial increase in $\varepsilon_{l}\;$ with Ag loading (8.2 → 193.1 → 108.6 → 469.8) indicates that Ag does not only contribute free carriers; it also enhances the background polarizability (bound-electron contribution), likely through an increased electronic density of states and strong interfacial polarization at Ag/PANI boundaries. The simultaneous elevation of $\varepsilon_{l}\;$ and $\frac{N}{m^{*}}\;$ implies that both polarization storage (intercept) and carrier dispersion (slope term) are reinforced. The relatively large *ε_l_* values obtained for the Ag/PANI films should be interpreted with caution. In the present work, *ε_l_* is not treated as a simple static dielectric constant of a homogeneous polymeric material, but as an effective model-derived parameter obtained from the optical dispersion analysis. Therefore, its magnitude can be strongly influenced by interfacial polarization, free-carrier contribution, structural heterogeneity, and fitting sensitivity, particularly in metal-containing nanocomposite films. From this perspective, the increase in *ε_l_* with Ag incorporation is consistent with an enhanced polarization response within the Ag/PANI system; however, the absolute values should be regarded as effective optical parameters within the applied model rather than direct intrinsic dielectric constants. Similar unusually high effective dielectric parameters have also been reported in heterogeneous conductive-polymer/metal and highly polarizable nanocomposite systems, where strong interface-related effects play a dominant role. Overall, Figure 11 and Table 5 confirm that Ag NP incorporation strongly amplifies the free-carrier dispersion of $\varepsilon_{r}$ in PANI-based films. The high $\left(\frac{N}{m^{*}}\right)\;$ and *ω_p_* values for Ag/PANI—15 wt.% (98.0 × 10^39^ and 248 × 10^12^ Hz) identify this composition as the most conductive/metal-like in optical dielectric behavior, while Ag/PANI—5 wt.% already exhibits a substantial enhancement ($\left(\frac{N}{m^{*}}\right)$ = 27.0 × 10^39^; *ω_p_* = 78.2 × 10^12^ Hz) with a large $\varepsilon_{l}\;$= 193.1. The non-monotonic values at 10 wt.% highlight that the microstructure and connectivity govern the effective carrier response, and should be considered when optimizing the films for optical and optoelectronic applications requiring tailored dielectric dispersion.

**Figure 12:**
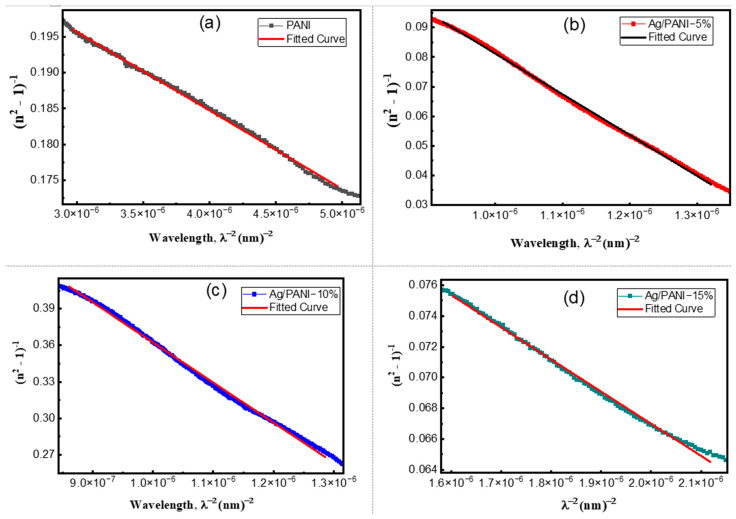
Plot of (*n*^2^ − 1)^−1^ versus squared wavelength (*λ*^2^) for (**a**) PANI and Ag/PANI nanocomposites: (**b**) 5%, (**c**) 10%, and (**d**) 15% Ag. Symbols represent experimental data, and solid lines indicate linear fitting, confirming normal dispersion behavior and Ag-dependent optical response.

Figure 12a–d presents the plot of ${\left(n^{2}\;-\;1\right)}^{-1}$ as a function of $\lambda^{-2}$ for pristine PANI and Ag/PANI nanocomposites. This representation is used to evaluate normal dispersion within a single-oscillator (Sellmeier-type) approach, where the refractive index is controlled by an effective electronic resonance located at *λ*_0_ and weighted by an oscillator strength $S_{0}$. The near-linear behavior in each panel indicates that the selected spectral range can be described by one dominant effective oscillator, allowing $S_{0}$ and *λ*_0_ to be extracted from the linear fit. The fitting relation is

(24) $${\left(n^{2}-1\right)}^{-1}=\frac{1}{S_{0}\lambda_{0}^{2}}-\left(\frac{1}{S_{0}}\right)\lambda^{-2}$$

Equation (24) predicts a straight line with a negative slope (−$\frac{1}{S_{0}}$) and intercept ($\frac{1}{S_{0}\lambda_{0}^{2}}$). Therefore, smaller $S_{0}$ produces a larger-magnitude slope (steeper dependence on $\lambda^{-2}$), while a larger *λ*_0_ tends to reduce the intercept for a fixed $S_{0}$. Table 5 reports the extracted S_0_ and *λ*_0_ values for all compositions. PANI exhibits $S_{0}$ = 9.1 × 10^−5^ m^−2^ and *λ*_0_ = 220 nm. From Equation (24), these values correspond to |−$\;\frac{1}{S_{0}}$| = 1.099 × 10^4^ m^2^ and an intercept $\frac{1}{S_{0}\lambda_{0}^{2}}$ = 0.2270. The moderate slope is consistent with a polymeric matrix showing a smooth normal dispersion. The 5 wt.% composite shows a strong red-shift of the characteristic wavelength to *λ*_0_ = 790 nm (3.5909× PANI) together with a sharp reduction in oscillator strength to $S_{0}$ = 0.7 × 10^−5^ m^−2^ (0.0769× PANI). Consequently, the slope magnitude increases to |−$\;\frac{1}{S_{0}}$| = 1.4286 × 10^5^ m^2^, which is 13.00× higher than PANI. Notably, the intercept remains close to the PANI value (0.2289 versus 0.2270), changing by only +0.0019 (+0.82%), because the decrease in $S_{0}$ is largely compensated by the increase in $\lambda_{0}^{2}$ in the intercept term. The 10 wt.% film exhibits the smallest oscillator strength, $S_{0}$ = 0.3 × 10^−5^ m^−2^ (0.0330× PANI), and a large *λ*_0_ = 690 nm (3.1364× PANI). This combination yields the steepest dispersion line in the series, with |−$\;\frac{1}{S_{0}}$| = 3.3333 × 10^5^ m^2^ (30.33× PANI). In addition, the intercept rises markedly to 0.7001, i.e., 3.08× PANI, indicating that the reduction in $S_{0}$ is not fully offset by $\lambda_{0}^{2}\;$ for this composition. The 10 wt.% sample therefore displays the strongest effective dispersion within the Equation (24) fit, consistent with its exceptionally large |−$\;\frac{1}{S_{0}}$| value. At 15 wt.% Ag, $S_{0}$ increases to 4.8 × 10^−5^ m^−2^ (0.5275× PANI) while *λ*_0_ decreases to 440 nm (2.000× PANI). As a result, the slope magnitude drops to |− $\frac{1}{S_{0}}$| = 2.0833 × 10^4^ m^2^, only 1.8958× higher than PANI and far smaller than the 5 and 10 wt.% cases.

The intercept decreases to 0.1076, which is 0.474× the PANI value. This indicates that high Ag loading partially restores the effective oscillator strength (larger $S_{0}$), reducing the steepness of the $\lambda^{-2}$ dispersion, while maintaining a resonance shifted to longer wavelengths than pristine PANI. The extracted *λ*_0_ values evolve non-monotonically with Ag NP loading: 220 nm (PANI) → 790 nm (5 wt.%) → 690 nm (10 wt.%) → 440 nm (15 wt.%). This signifies a pronounced red-shift of the effective dispersion resonance at intermediate loadings, followed by a partial blue-shift at the highest loading. In parallel, $S_{0}$ decreases strongly at 5 wt.% (−92.31% relative to PANI) and 10 wt.% (−96.70%), then increases at 15 wt.% (4.8 × 10^−5^ m^−2^) but remains below PANI (−47.25%). Since the slope equals −$\;\frac{1}{S_{0}\;}$, these values explain the steepness ranking directly: 10 wt.% (steepest) > 5 wt.% ≫ 15 wt.% > PANI. Overall, Figure 12 and Table 5 demonstrate that Ag addition tunes the dispersion behavior by jointly altering oscillator strength ($S_{0}$) and resonance position (*λ*_0_). The strong $S_{0}$ suppression at 5–10 wt.% implies that the effective single-oscillator contribution becomes weaker, even while *λ*_0_ shifts to much longer wavelengths, consistent with Ag-induced electronic coupling and interfacial polarization effects. At 15 wt.%, the recovery of $S_{0}$ together with a smaller *λ*_0_ suggests a change in the electronic environment and connectivity (e.g., stronger metal-like interaction and altered percolation), leading to a less steep but still Ag-modified dispersion response.

### 3.6. FTIR Spectra

**Figure 13:**
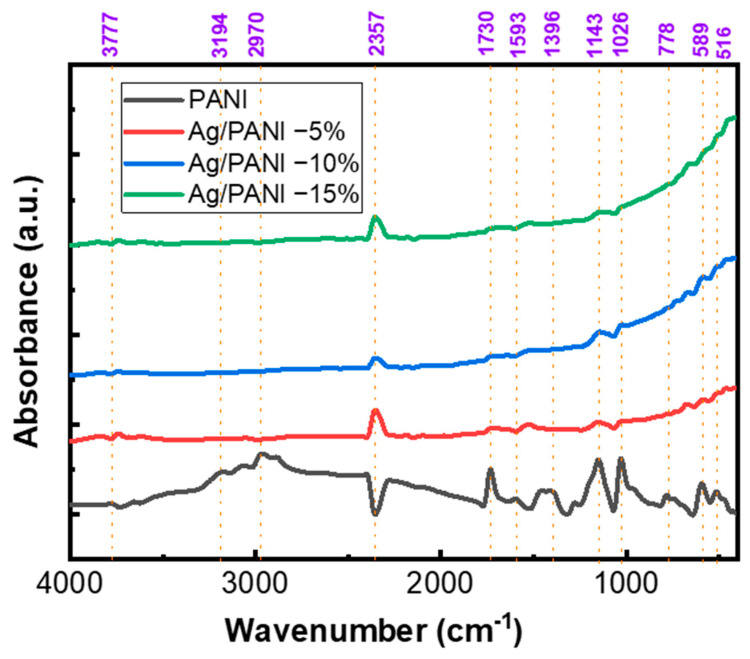
FTIR absorbance spectra of pristine PANI and Ag/PANI. Characteristic bands at 3777, 3194, 2970, 2357, 1730, 1593, 1386, 1143, 1026, 778, 589, and 516 cm^−1^ highlight O-H/N–H stretching, backbone ring and C-N vibrations, doped-state sensitive modes, and low-frequency interfacial features that evolve with Ag loading.

Figure 13 shows the FTIR absorbance spectra of pristine PANI and Ag/PANI nanocomposite films (5, 10, and 15 wt.% Ag) collected over the 4000–400 cm^−1^ range. Presenting the spectra in absorbance highlights the evolution of vibrational band intensities with Ag incorporation, where larger peak heights correspond to stronger infrared absorption. The labeled wavenumbers (3777, 3194, 2970, 2357, 1730, 1593, 1386, 1143, 1026, 778, 589, and 516 cm^−1^) provide a quantitative guide for band assignment and compositional comparison. In the high-wavenumber region, the weak feature at 3777 cm^−1^ is consistent with O–H stretching from surface hydroxyl groups or weakly bound moisture. The broad band centered around 3194 cm^−1^ is characteristic of N–H stretching in PANI (amine/imine units), typically broadened by hydrogen bonding. Compared with pristine PANI, the Ag/PANI spectra show this region as a smoother baseline with a reduced fine structure, suggesting that Ag addition modifies the local hydrogen-bonding environment and the effective dipole-moment change in N–H vibrations. A smaller contribution near 2970 cm^−1^ is attributable to C–H stretching (aliphatic/aryl), which can include residual organic species from processing [52]. The distinct band around 2357 cm^−1^ is commonly associated with atmospheric CO_2_ absorption (asymmetric stretching). Its presence across the spectra is therefore treated as an external contribution rather than a chemical fingerprint of the films. In the carbonyl region, the absorption at 1730 cm^−1^ indicates a C=O stretching component. In PANI-based systems, this can arise from minor oxidative functionalities, residual dopant/solvent-derived groups, or interfacial species generated during film growth. The emergence of a measurable 1730 cm^−1^ contribution in the Ag/PANI films suggests that Ag incorporation increases the population or IR activity of carbonyl-type groups, consistent with a more reactive interfacial environment [53].

The aromatic backbone region provides the primary evidence that the PANI structure is retained in the nanocomposites. The band at 1593 cm^−1^ lies within the range of quinoid/benzenoid ring stretching vibrations in PANI. Changes in the intensity and sharpness of this feature with Ag loading indicate altered conjugation and chain packing. The band at 1386 cm^−1^ is consistent with C–N stretching and/or ring deformation modes; in doped PANI, this region can also be influenced by counter-ion interactions, so its persistence supports that the polymer remains in a doped/protonated form [54]. A prominent absorption at 1143 cm^−1^ is widely recognized in doped PANI as an “electronic-like” band associated with C–N^+^ stretching coupled to charge delocalization (polaron/bipolaron states). The clear presence of the 1143 cm^−1^ band in the Ag/PANI spectra indicates that Ag incorporation does not suppress the conducting-state vibrational signature; rather, it can enhance local polarization and charge-transfer coupling at the Ag–PANI interface. The neighboring feature at 1026 cm^−1^ is attributed to in-plane C–H bending and/or C–N stretching contributions; variations in this band are sensitive to dopant configuration and local chain conformation, providing further evidence that Ag affects the polymer–dopant microenvironment [55]. In the low-wavenumber region, the band at 778 cm^−1^ corresponds to out-of-plane aromatic C–H deformation, while the features at 589 and 516 cm^−1^ are assigned to ring deformation modes and/or interfacial vibrations involving metal–nitrogen coordination or metal-oxide/hydroxide surface layers on Ag NPs. The presence and strengthening of these low-frequency bands in the composites is consistent with additional vibrational contributions introduced by the nanoparticle phase and its interface with the polymer matrix [56]. Overall, the spectra confirm that (***i***) the PANI backbone vibrations remain present after Ag incorporation (retention of the 1593, 1386, 1143, and 1026 cm^−1^ regions), and (***ii***) Ag NP loading modifies the spectral envelope and relative band intensities, especially in the doped-state sensitive band at 1143 cm^−1^ and the low-frequency region (589–516 cm^−1^). The systematic labeling of peaks at 3777, 3194, 2970, 2357, 1730, 1593, 1386, 1143, 1026, 778, 589, and 516 cm^−1^ provides direct quantitative markers for tracking compositional trends and supports the conclusion that Ag nanoparticles introduce stronger interfacial interactions and enhanced local polarization within Ag/PANI nanocomposite films [57,58,59,60].

### 3.7. Electrical Conductivity

Electrical (DC) conductivity was measured for pristine polyaniline (PANI) and Ag/PANI nanocomposites. The measured conductivities reveal a systematic enhancement of charge transport upon incorporation of Ag nanoparticles, indicating that metallic inclusions and polymer–metal interfacial coupling effectively strengthen the conductive network within the film. Pristine PANI exhibits a baseline conductivity of *σ* = 1.0 S·cm^−1^. This value reflects charge transport dominated by polaron/bipolaron carriers along conjugated segments with interchain hopping across disordered regions. The moderate conductivity of the polymer matrix sets the reference for evaluating the impact of Ag loading. Upon adding 5 wt.% Ag, conductivity increases to *σ* = 2.6 S·cm^−1^. This corresponds to a 2.60× enhancement relative to pristine PANI and an absolute gain of Δ*σ* = +1.6 S·cm^−1^ (i.e., a +160% increase). At this loading, Ag nanoparticles are expected to introduce additional conductive islands and locally enhance carrier mobility by strengthening chain packing and facilitating charge transfer at Ag-PANI interfaces. Even if a fully continuous Ag-Ag percolation path is not yet formed, the reduced effective hopping distance between conducting domains can substantially decrease the overall resistance. Further increasing Ag to 10 wt.% yields *σ* = 4.2 S·cm^−1^, representing a 4.20× enhancement and Δ*σ* = +3.2 S·cm^−1^ (+320%) compared with PANI. Relative to the 5 wt.% film, conductivity rises by 1.6 S·cm^−1^ (from 2.6 to 4.2 S·cm^−1^), indicating that the increased nanoparticle population promotes improved connectivity among conductive regions. This behavior is consistent with the development of more frequent interparticle junctions and a higher density of polymer–metal interfaces, both of which facilitate current flow across the film plane. The highest Ag loading (15 wt.%) produces the maximum conductivity, *σ* = 7.0 S·cm^−1^. This corresponds to 7.00× the PANI value, with Δ*σ* = +6.0 S·cm^−1^ (+600%). Compared with the 10 wt.% film, σ increases by 2.8 S·cm^−1^ (from 4.2 to 7.0 S·cm^−1^), which is a +66.7% increment. The stronger rise between 10 and 15 wt.% suggests that the composite approaches a more effective percolative transport regime, where Ag-assisted pathways contribute more directly to DC conduction. In this regime, current is expected to propagate through a hybrid network consisting of conducting PANI domains bridged by closely spaced Ag NPs, reducing the dominance of hopping-limited polymer transport. From an electrical-transport standpoint, the monotonic increase in σ across 0–15 wt.% Ag indicates that, within the investigated range, the benefits of increased connectivity and interfacial charge-transfer outweigh possible adverse effects such as nanoparticle agglomeration or junction barrier formation. Nevertheless, the absolute conductivity remains below that of bulk metals, confirming that the films behave as polymer-dominated composites rather than continuous metallic layers. Overall, the Ag/PANI nanocomposites demonstrate a clear improvement in DC conductivity with Ag incorporation: 2.6 S·cm^−1^ (5 wt.%), 4.2 S·cm^−1^ (10 wt.%), and 7.0 S·cm^−1^ (15 wt.%) compared with 1.0 S·cm^−1^ for pristine PANI. These results indicate that controlling Ag loading is an effective route to tune electrical transport in PANI-based thin films for applications requiring enhanced conductivity.

### 3.8. Surface Morphology

**Figure 14:**
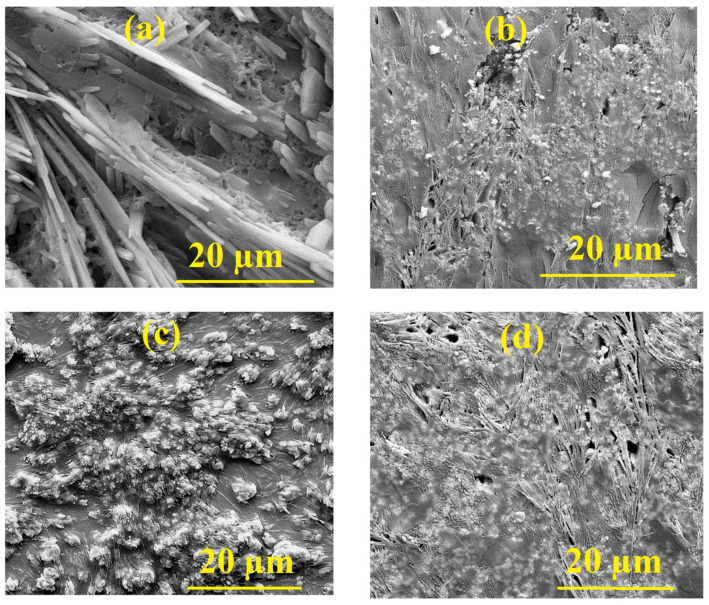
SEM micrographs of electrochemically deposited PANI and Ag/PANI films with increasing Ag NP content: (**a**) pristine PANI (fibrous/lamellar), (**b**) 5 wt.% Ag/PANI (granular, more heterogeneous), (**c**) 10 wt.% Ag/PANI (denser particulate/nodular), and (**d**) 15 wt.% Ag/PANI (highest heterogeneity/agglomeration). All images are at the same magnification (scale bar: 20 μm).

The pristine PANI film (Figure 14a) exhibits a distinctly anisotropic, fibrous/lamellar texture, with elongated features arranged in partially aligned bundles. This morphology is consistent with electro-polymerization dominated by directional growth and coalescence of polymer domains, yielding a surface with pronounced micro-scale ridges and inter-fiber gaps. Such a structure typically provides a relatively high external surface area while maintaining a continuous polymer network, which is beneficial for electronic conduction along interconnected pathways. Upon introducing a low-Ag NP fraction (Figure 14b), the surface becomes more heterogeneous and less uniformly fibrillar. The micrograph indicates the development of fine granular contrast superimposed on the polymer background, suggesting that Ag NPs (and/or NP-rich domains) are embedded within or anchored onto the PANI matrix. At this loading, the polymer still appears to form a continuous scaffold, while the nanoparticles plausibly increase nucleation density and disrupt long-range fiber alignment. The overall morphology suggests a transition from purely polymer-controlled growth to composite-assisted nucleation, without severe clustering. At intermediate loading (Figure 14c), the surface evolves toward a more densely textured and particulate-rich appearance. Compared with 5 wt.%, the microstructure shows larger aggregated features and a more pronounced “clustered” topography, indicating that nanoparticle–nanoparticle interactions increasingly compete with polymer encapsulation. This regime typically corresponds to a morphology where the polymer matrix still bridges domains, but the surface becomes dominated by composite nodules and a higher density of protrusions, which can increase the effective area yet also introduce stronger spatial variations in local conductivity and interfacial potential. At the highest Ag content (Figure 14d), the film displays the most irregular and defect-prone surface, with conspicuous heterogeneity and features consistent with agglomeration and nonuniform coalescence. The polymer texture appears less clearly fibrous and more disrupted, implying that excessive particle content can hinder uniform polymer growth and promote stress accumulation during deposition and drying. This morphology can create mixed outcomes: it may increase surface roughness and potential active sites, but it can also reduce film uniformity and continuity, promoting microvoids or weakly connected domains that adversely affect reproducibility and long-term stability.

The SEM series supports a coherent mechanism in which Ag NPs initially act as additional nucleation/anchoring sites within the growing PANI, leading to moderated disruption of polymer alignment (5 wt.%). As loading increases (10–15 wt.%), particle–particle interactions become more influential, favoring cluster formation and a more heterogeneous composite texture. This progression is important because it directly affects interfacial and transport behavior. First, rough, clustered surfaces typically provide a higher density of accessible sites, which can enhance interfacial processes.

However, excessive clustering may compromise transport uniformity and percolation by producing spatially nonuniform conduction pathways and localized barriers, particularly if polymer bridging becomes insufficient. Second, highly heterogeneous growth can concentrate stress, and thereby weaken film cohesion and disrupt continuity. Overall, SEM confirms that Ag incorporation does not merely “decorate” the PANI surface; rather, it restructures the growth morphology in a loading-dependent manner. These microstructural findings provide a critical basis for correlating the Ag content with the film’s functional properties in later sections [61,62].

### 3.9. XRD Analysis

**Figure 15:**
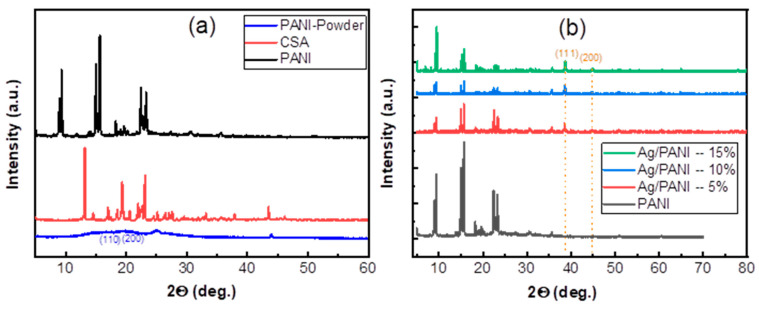
XRD patterns of (**a**) PANI, CSA, and PANI powder, and (**b**) pure PANI and Ag/PANI nanocomposite films with 5, 10, and 15 wt.% Ag. The Ag/PANI films show the emergence and growth of the fcc Ag peaks at 2*θ* ≈ 38.1° and 44.3°, assigned to the (111) and (200) planes, confirming successful Ag incorporation into the PANI matrix.

The pristine PANI film exhibits a semi-crystalline fingerprint consisting of broad reflections at 2*θ* = 9.29°, 15.63°, 18.28°, 22.56°, and 27.43°(Figure 15). These features are commonly associated with short-range ordering of the emeraldine salt form of PANI and periodic packing of polymer chains. Using Cu K_α_ radiation (*λ* = 1.5406 Å), the corresponding interplanar spacings are d = 9.513, 5.665, 4.848, 3.938, and 3.250 Å, respectively (Table A2). The reflections centered near ~18–23° can be indexed to the (110) and (200) family reported for partially ordered PANI domains; in the present film, the (110)-type peak at 2*θ* = 18.28° exhibits FWHM = 0.115° (Scherrer D ≈ 73.3 nm), while the (200)-type peak at 2*θ* = 22.56° has FWHM = 0.136° (D ≈ 62.3 nm). The breadth of these peaks confirms that ordering is limited to nanoscale coherence lengths rather than long-range crystallinity. Upon incorporating Ag nanoparticles, the PANI-related features remain present but undergo subtle shifts and broadening changes, indicating that metal loading perturbs chain packing and inter-chain spacing. In addition, distinct Bragg peaks emerge at 2*θ* = 38.63–38.78° and 44.82–44.97°, which are assigned to the (111) and (200) reflections of face-centered cubic (fcc) metallic Ag. The measured d-spacings for Ag (111) decrease slightly from 2.329 Å (5 wt.% Ag, 2*θ* = 38.63°) to 2.320 Å (15 wt.% Ag, 2*θ* = 38.78°), consistent with a small lattice contraction or interfacial strain within the polymer matrix. From these reflections, the corresponding fcc lattice parameter is a ≈ 4.02–4.04 Å (calculated from d111√3 and 2d200), which is close to the bulk value expected for Ag and supports the metallic phase assignment. The Ag peaks become more evident with increasing Ag loading, while no additional reflections attributable to silver oxides are apparent within the detection limit of the plotted data, suggesting that the deposited nanoparticles are predominantly metallic. Line-broadening analysis further indicates that Ag is present as nanocrystalline domains. For the Ag (111) peak, the extracted FWHM values are 0.196°, 0.189°, and 0.170° for 5, 10, and 15 wt.% Ag, corresponding to Scherrer crystallite sizes of 45.1, 46.5, and 51.8 nm, respectively. Within the uncertainty of plot-based digitization, these values indicate an Ag crystallite size in the order of ~10 nm across the composition window, with peak sharpening and intensity growth primarily reflecting a higher phase fraction and improved diffracting volume rather than a dramatic change in size. Meanwhile, the PANI (110)/(200) peaks show composition-dependent shifts (e.g., the (200)-type peak moves from 22.56° in PANI/low-Ag films to 22.85° at 15 wt.% Ag), consistent with modest contraction of the average inter-chain spacing (d decreases from 3.938 to 3.888 Å). Overall, the XRD results confirm the successful incorporation of fcc Ag nanoparticles into the PANI matrix while preserving the polymer’s semi-crystalline signature.

#### Williamson–Hall (W–H) Microstructural Analysis

**Figure 16:**
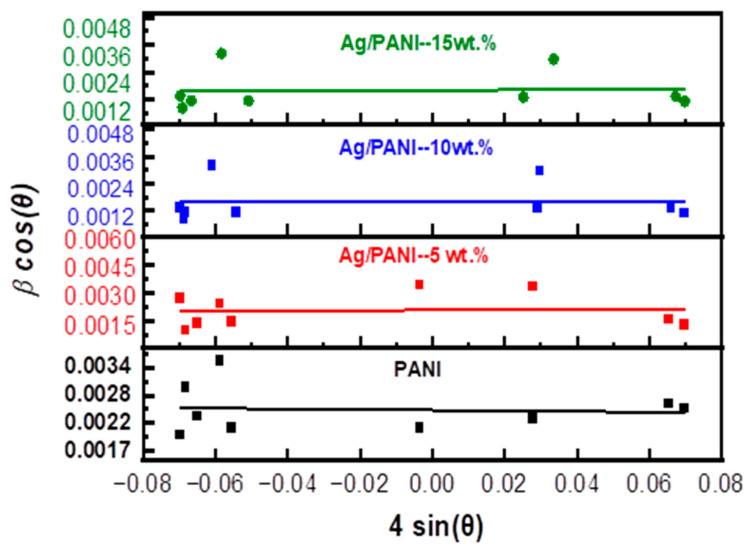
W-H plot of PANI and Ag/PANI nanocomposites.

The crystallographic broadening of diffraction peaks in semicrystalline conducting polymers and their metal–polymer nanocomposites is commonly governed by the combined effects of a finite coherent domain size and lattice distortions (microstrain). To separate these two contributions for the present thin films, the Williamson–Hall (W–H) approach was employed using the uniform deformation model. The analysis is based on the linearized relation:

(25) $$\beta_{s\;\;}cos(\theta )=\varepsilon \;(4\;sin(\theta ))+\;\frac{K\lambda }{D}$$

where *β* is the full width at half maximum (FWHM) expressed in radians after converting from degrees, *θ* is the Bragg angle, *K* is the Scherrer shape factor, *λ* is the X-ray wavelength (Cu K*α*), *D* is the average crystalline grain size (coherent domain size), and *ε* is the microstrain. Accordingly, a plot of *β cos*(*θ*) versus 4 *sin*(*θ*) yields a straight line whose intercept equals *Kλ/D* (allowing extraction of *D*) and whose slope corresponds to the microstrain *ε* (Figure 16). The resulting microstructural parameters for pristine PANI and Ag/PANI films with 5–15 wt.% Ag are summarized in Table 6. For pristine PANI, W–H fitting gives an average crystalline grain size of *D* = 58 nm together with a microstrain of *ε* = 6.47 × 10^−4^. The corresponding dislocation density, estimated from the usual inverse-square dependence on crystallite size (*δ ≈* 1*/D*^2^), is *δ* = 29.7 × 10^13^ m^−2^, indicating a comparatively defect-rich microstructure typical of electrochemically grown PANI where chain packing is locally disrupted by dopant counter-ions and polymerization-induced disorder. Upon incorporation of Ag NPs, the coherent domain size increases markedly. At 5 wt.%, *D* rises to 69 nm, while at 10 wt.% and 15 wt.%, it reaches 91 nm and 92 nm, respectively. This monotonic increase in *D* with Ag NP loading indicates that Ag NP incorporation promotes improved structural coherence of the PANI matrix, plausibly by facilitating more effective chain organization and by reducing the density of size-limiting defects through heterogeneous nucleation and enhanced local ordering around the metallic inclusions.

In contrast to the steady grain-growth trend, the lattice strain exhibits a non-monotonic dependence on Ag content, reflecting the competing roles of interfacial mismatch and strain relaxation through coarsening. Specifically, the microstrain increases from 6.47 × 10^−4^ (PANI) to 10.8 × 10^−4^ at 5 wt.%, suggesting that initial Ag insertion introduces localized stress fields at Ag/PANI interfaces and/or perturbs the polymer lattice through mismatch in thermal/elastic response. At higher loading, however, the strain relaxes strongly: *ε* decreases to 2.8 × 10^−4^ at 10 wt.%, the lowest value among all samples, before rising moderately to 5.6 × 10^−4^ at 15 wt.%. The minimum strain at 10 wt.% indicates that this composition achieves the most relaxed microstructure, where the benefits of increased coherence length and improved packing outweigh the strain introduced by additional interfaces. The slight re-increase at 15 wt.% can be attributed to the higher interfacial area and possible nanoparticle–nanoparticle proximity effects, which may reintroduce lattice distortions even though *D* remains high. Consistent with the grain-size evolution, the dislocation density decreases substantially with Ag NP addition. *δ* decreases from 29.7 × 10^13^ m^−2^ for PANI to 21.9 × 10^13^ m^−2^ at 5 wt.% and reaches 12.2 × 10^13^ m^−2^ and 12.1 × 10^13^ m^−2^ at 10 and 15 wt.%, respectively. The nearly identical *δ* values for 10 and 15 wt.%, together with the saturation of *D* near ~92 nm, suggest that the defect suppression and ordering improvement approach a plateau beyond ~10 wt.%. In other words, once a sufficiently coherent polymer–metal network is established, further Ag incorporation contributes less to increasing the coherent domain size and mainly modulates strain through interfacial effects.

To further link macrostrain to stored elastic energy, the strain energy density was estimated using the commonly adopted expression (Equation (26)) [62,63].

*U_s_* = 1/2 *E ε*^2^ (26)

where *E* is Young’s modulus and *ε* is the microstrain in absolute units. Using *E* = 2.5 GPa as a representative modulus for PANI-based films, the calculated strain energy densities are *U_s_* = 5.233 × 10^+2^ J/m^3^ (PANI), 1.458 × 10^+2^ J/m^3^ (5 wt.%), 9.800 × 10^+1^ J/m^3^ (10 wt.%), and 3.920 × 10^+2^ J/m^3^ (15 wt.%). The *U_s_* trend follows the strain evolution: the highest energy at 5 wt.% reflects the elevated microstrain, whereas the minimum at 10 wt.% confirms this film as the most strain-relaxed despite its large grain size. The moderate increase at 15 wt.% again highlights the contribution of interfacial strain as Ag loading rises.

Overall, the W–H analysis demonstrates that Ag NP incorporation significantly enhances crystalline coherence in PANI thin films (*D* increases from 58 nm to ~92 nm) and suppresses defect density (*δ* decreases from 29.7 × 10^13^ m^−2^ to 12.1 × 10^13^ m^−2^). Meanwhile, microstrain and the associated elastic energy display a non-monotonic response with a pronounced optimum at 10 wt.%, where the film combines a large coherent domain size with the lowest microstrain and stored energy. These microstructural outcomes are consistent with a transition from interface-dominated distortions at low Ag loading to strain-accommodated, structurally stabilized nanocomposite films at intermediate loading, followed by renewed interfacial distortion at the highest Ag NP fraction.

Table 7 highlights that the Ag filled PANI films in this work behave very differently from previously reported PANI/oxide nanocomposites. Here, Ag incorporation drives a strong reduction in the indirect bandgap from 1.98 eV (PANI) to 1.81 eV (5 wt.% Ag), 1.38 eV (10 wt.% Ag), and 1.19 eV (15 wt.% Ag), placing the system in a distinctly low-gap regime. In contrast, oxide-filled PANI systems (NiO, TiO_2_, NiFe_2_O_4_, Al_2_O_3_) show much larger gaps of 3.45–3.79 eV, consistent with the wide-bandgap nature of these fillers and stronger carrier localization. The Urbach energy in this work increases sharply from 377 meV to 1280–1640 meV at 5–10 wt.% Ag, indicating broad tail states and enhanced energetic disorder; by comparison, oxide composites exhibit relatively low EU (220–470 meV). Structurally, the strain in Ag/PANI is non-monotonic (10.8 × 10^−4^ → 2.8 × 10^−4^ → 5.6 × 10^−4^), suggesting competing interfacial distortion and relaxation, whereas oxide systems mostly remain within the ~10^−4^ range.

## 4. Conclusions

This work establishes a composition-resolved understanding of how Ag NPs restructure the electronic and optical response of electrodeposited PANI thin films on ITO, addressing a persistent gap in the literature where band-edge disorder, dispersion energetics, dielectric carrier effects, and *χ*^3^ are typically treated separately. Using a single, controllable fabrication route (potentiostatic electrodeposition with 5–15 wt.% Ag in the electrolyte), the films provide a consistent platform to isolate loading-dependent trends relevant to integrated optoelectronics. Key outcomes can be summarized as follows. (***i***) Electronic-structure tuning is accompanied by strong disorder broadening: Ag addition narrows the indirect gap from 1.98 eV (pristine PANI) to 1.81, 1.38, and 1.19 eV at 5, 10, and 15 wt.% Ag, respectively, while the Urbach energy rises sharply to 1.28–1.64 eV at 5–10 wt.%, indicating a substantial increase in localized tail states and electron–phonon coupling signatures (with Urbach fitting becoming unreliable at 15 wt.% under strong loss conditions). (***ii***) Dispersion parameters evolve non-monotonically, revealing that the Ag fraction alone is not predictive: the Wemple–DiDomenico analysis shows that the dispersion energy and oscillator energy shift in different directions across the series, consistent with competing effects of interfacial polarization, morphology, and redistribution of oscillator strength. (***iii***) The dielectric response transitions toward a carrier-rich regime at high loading: Drude-type analysis indicates large increases in *ε_∞_*, N/m*, and *ω_p_*, reaching *ε_∞_* ≈ 469.8 and *ω_p_* ≈ 248 × 10^12^ Hz for 15 wt.% Ag, evidencing pronounced long-wavelength polarization and free-carrier contributions. (***iv***) Third-order nonlinearity is strongly enhanced yet highly sensitive to the microstructure: *χ*^3^ increases by more than an order of magnitude at 5 and 15 wt.% (≈7.6 × 10^−8^ esu), but the Kerr coefficient *n*_2_ is maximized at 5 wt.% (25.91 × 10^−7^ esu) and suppressed at 10 wt.% and 15 wt.% despite a large *χ*^3^, implying loss/dispersion and connectivity effects that diminish effective Kerr refraction. Overall, the central conclusion is that Ag/PANI optical performance is governed by a non-monotonic disorder–dispersion coupling: moderate loading (5 wt.%) provides the most favorable balance of interfacial polarization and manageable loss, producing the best Kerr-type response, whereas higher loading increases carrier-driven dielectric dispersion but can shift the nonlinear response toward dissipative channels and reduce effective *n*_2_. These findings offer a quantitative basis for selecting Ag loading according to target functionality-maximizing Kerr switching at moderate loading versus maximizing carrier-rich dielectric behavior at high loading, and motivate future work that directly links nanoparticle connectivity (e.g., percolation metrics and spatial dispersion state) to a nonlinear figure-of-merit under device-operating wavelengths and intensities.

## Figures and Tables

**Table 1:** Comparative analysis of electronic structural parameters in PANI and nanocomposites.

Sample	Indirect Energy Gap (*E_g_*) (eV)	Urbach Energy (meV)	Steepness Parameter (γ) (eV^−1^)	Strength of Electron–Phonon (*E_e_p_*) (eV)
PANI	1.98	377	0.06	10.7
Ag/PANI—5 wt.%	1.81	1280	0.02	36.2
Ag/PANI—10 wt.%	1.38	1640	0.01	46.4
Ag/PANI—15 wt.%	1.19	Not reliable	***	***

**Table 2:** Estimated birefringence and extraordinary refractive index.

Sample	Average Refractive Index (*n̄* )	Birefringence (Δ*n*)	Estimated *n*_e_ (max *n*)	Estimated *n*_0_ (min *n*)
PANI	2.228	0.475	2.545	2.070
Ag/PANI—5 wt.%	5.133	4.591	8.194	3.603
Ag/PANI—10 wt.%	2.924	2.975	4.907	1.932
Ag/PANI—15 wt.%	6.556	9.410	12.829	3.419

**Table 3:** The values of *E_d_*, *E*_0_, *f*, *n*_0_, *ε_s_*, *M*_−1_, and *M*_−3_.

Sample	*E_d_* (eV)	*E*_0_ (eV)	*f* (eV)^2^	*n*_0_	*ε_s_*	*M*_−1_ (ev)^−2^	*M*_−3_ (ev)^−2^
PANI	4.81	1.92	9.23	1.18	1.40	2.51	0.68
Ag/PANI—5 wt.%	7.19	1.56	11.25	1.10	1.22	4.60	1.88
Ag/PANI—10 wt.%	2.58	1.79	4.63	1.30	1.69	1.44	0.45
Ag/PANI—15 wt.%	24.3	5.27	128.21	1.10	1.22	4.60	0.17

**Table 4:** The *χ*^1^, *χ*^3^ and *n*_2_ values of PANI and Ag/PANI nanocomposites.

Sample	*χ* ^1^	*χ*^3^ (e.s.u. × 10^−9^)	*n*_2_ (e.s.u. × 10^−7^)
PANI	0.2	6.73	2.15
Ag/PANI—5 wt.%	0.4	75.82	25.91
Ag/PANI—10 wt.%	0.1	0.73	0.21
Ag/PANI—15 wt.%	0.4	76.56	0.26

**Table 5:** Optical parameters of PANI and Ag/PANI, including N/m*, *εₗ*, *ω_p_*, *S*_0_, and *λ*_0_ values.

Sample	$\frac{N}{m^{*}}\times 10^{39}$ $\left({kg}^{-1}m^{-3}\right)$	${ɛ}_{l}$	$\omega_{p}\times 10^{12}$ (Hz)	$S_{0}\times 10^{-5}$ $\;(m^{-2})$	$\lambda_{0}$ (nm)
PANI	1.6	8.2	4.8	9.1	220
Ag/PANI—5 wt.%	27.0	193.1	78.2	0.7	790
Ag/PANI—10 wt.%	21.7	108.6	62.9	0.3	690
Ag/PANI—15 wt.%	98.0	469.8	248	4.8	440

**Table 6:** Extracted crystallographic features of PANI-based nanocomposites, including crystallite size, dislocation density, lattice strain, and strain energy density.

Sample	Average Crystalline Grain Size (*D*) (nm)	Strain (*ε* × 10^−4^)	Dislocation Density (*δ* × 10^13^ m^−2^)	Strain Energy Density (*U_s_*) (J/m^3^) [62,63]
PANI	58	6.47	29.7	5.23 × 10^2^
Ag/PANI—5 wt.%	69	10.8	21.9	1.46 × 10^3^
Ag/PANI—10 wt.%	91	2.8	12.2	9.80 × 10^1^
Ag/PANI—15 wt.%	92	5.6	12.1	3.92 × 10^2^

**Table 7:** Optical and structural parameters of PANI and its nanocomposites: This work versus selected published studies.

Sample	Ref.	Indirect Energy Gap (*E*_*g*_) (eV)	Urbach Energy (meV)	Strain (*ε*)
PANI	This work	1.98	377	6.47 × 10^−4^
Ag/PANI—5 wt.%	This work	1.81	1280	10.8 × 10^−4^
Ag/PANI—10 wt.%	This work	1.38	1640	2.8 × 10^−4^
Ag/PANI—15 wt.%	This work	1.19	Not reliable	5.6 × 10^−4^
PANI/NiO—5 wt.%	[64]	3.45	290	13.5 × 10^−4^
PANI/TiO_2_—10 wt.%	[64]	3.53	240	5.90 × 10^−4^
PANI/NiFe_2_O_4_—15 wt.%	[65]	3.60	220	5.00 × 10^−4^
PANI/Al_2_O_3_—5 wt.%	[56]	3.76	463	3.6 × 10^−4^
PANI/Al_2_O_3—_10 wt.%	[56]	3.79	470	4.9 × 10^−5^

## Data Availability

The original contributions presented in this study are included in the article. Further inquiries can be directed to the corresponding authors.

## Appendix A. Supporting Information and Data

### Appendix A.1. Atomic Force Microscopy (AFM)

**Table A1:** AFM roughness metrics extracted from the same 20 µm × 20 µm scan areas shown in Figure 2 (tapping mode; 512 × 512 pixels). Areal roughness parameters (S_a_, S_q_, S_z_; with R_z_ = z_max_ − z_min_). Values are reported in nm; S_z_/R_z_ is additionally reported in nm.

Sample	R_a_ (nm)	R_q_ (nm)	R_z_ (nm)
**PANI (pristine)**	240.088	303.869	2283.39
**Ag/PANI (15 wt** **.** **%)**	637	830	4740

### Appendix A.3. XRD Analysis

**Table A2:** **FWHM values (from Scherrer crystallite sizes)** calculated using the Scherrer relation D = K*λ*/(*β* cos*θ*) rearranged as *β* = K*λ*/(D cos*θ*), where *β* is the peak full width at half maximum (FWHM) in radians and *θ* = (2*θ*)/2. Assumes K = 0.90 and Cu K_α_ wavelength *λ* = 1.5406 Å. FWHM is reported in degrees. If your D values were instrument-corrected, these *β* values represent the corrected broadening term.

Sample	2*θ* (deg)	d (Å)	FWHM (deg)	Crystallite Size D (nm)	Phase	hkl
PANI film	9.29	9.513	0.0920	86.6	PANI (emeraldine salt)	
PANI film	15.63	5.665	0.1332	60.2	PANI (emeraldine salt)	
PANI film	18.28	4.848	0.1151	73.3	PANI (emeraldine salt)	(110)
PANI film	22.56	3.938	0.1361	62.3	PANI (emeraldine salt)	(200)
PANI film	27.43	3.250	0.1288	63.5	PANI (emeraldine salt)	
Ag/PANI 5 wt.%	9.44	9.365	0.0900	88.6	PANI (emeraldine salt)	
Ag/PANI 5 wt.%	15.63	5.665	0.1310	61.2	PANI (emeraldine salt)	
Ag/PANI 5 wt.%	18.43	4.810	0.0803	100.2	PANI (emeraldine salt)	(110)
Ag/PANI 5 wt.%	22.56	3.938	0.1537	52.7	PANI (emeraldine salt)	(200)
Ag/PANI 5 wt.%	38.63	2.329	0.0744	113.2	fcc Ag	(111)
Ag/PANI 5 wt.%	44.82	2.020	0.0948	90.6	fcc Ag	(200)
Ag/PANI 10 wt.%	9.29	9.513	0.0936	85.2	PANI (emeraldine salt)	
Ag/PANI 10 wt.%	15.78	5.613	0.1146	70.0	PANI (emeraldine salt)	
Ag/PANI 10 wt.%	18.43	4.810	0.1677	48.0	PANI (emeraldine salt)	(110)
Ag/PANI 10 wt.%	22.56	3.938	0.1750	46.3	PANI (emeraldine salt)	(200)
Ag/PANI 10 wt.%	27.57	3.232	0.1623	50.4	PANI (emeraldine salt)	
Ag/PANI 10 wt.%	38.63	2.329	0.0866	97.2	fcc Ag	(111)
Ag/PANI 10 wt.%	44.82	2.020	0.0846	101.6	fcc Ag	(200)
Ag/PANI 15 wt.%	9.44	9.365	0.0877	90.9	PANI (emeraldine salt)	
Ag/PANI 15 wt.%	15.78	5.613	0.1204	66.6	PANI (emeraldine salt)	
Ag/PANI 15 wt.%	18.58	4.772	0.1078	74.7	PANI (emeraldine salt)	(110)
Ag/PANI 15 wt.%	22.85	3.888	0.1326	61.1	PANI (emeraldine salt)	(200)
Ag/PANI 15 wt.%	27.72	3.216	0.0908	90.1	PANI (emeraldine salt)	
Ag/PANI 15 wt.%	38.78	2.320	0.0762	110.5	fcc Ag	(111)
Ag/PANI 15 wt.%	44.97	2.014	0.0810	106.1	fcc Ag	(200)
